# Sanitation and water supply coverage thresholds associated with active trachoma: Modeling cross-sectional data from 13 countries

**DOI:** 10.1371/journal.pntd.0006110

**Published:** 2018-01-22

**Authors:** Joshua V. Garn, Sophie Boisson, Rebecca Willis, Ana Bakhtiari, Tawfik al-Khatib, Khaled Amer, Wilfrid Batcho, Paul Courtright, Michael Dejene, Andre Goepogui, Khumbo Kalua, Biruck Kebede, Colin K. Macleod, Kouakou IIunga Marie Madeleine, Mariamo Saide Abdala Mbofana, Caleb Mpyet, Jean Ndjemba, Nicholas Olobio, Alexandre L. Pavluck, Oliver Sokana, Khamphoua Southisombath, Fasihah Taleo, Anthony W. Solomon, Matthew C. Freeman

**Affiliations:** 1 Department of Environmental Health, Rollins School of Public Health, Emory University, Atlanta, GA, United States of America; 2 Department of Public Health, Environmental and Social Determinants of Health, World Health Organization, Geneva, Switzerland; 3 International Trachoma Initiative, The Task Force for Global Health, Decatur, GA, United States of America; 4 Department of Ophthalmology, Sana'a University, Sana'a, Yemen; 5 Department of Ophthalmology, Ministry of Health, Cairo, Egypt; 6 Programme National de Lutte contre les Maladies Transmissibles, Ministère de la Santé, Cotonou, Bénin; 7 Division of Ophthalmology, Kilimanjaro Centre for Community Ophthalmology International, University of Cape Town, Cape Town, South Africa; 8 Michael Dejene Public Health Consultancy Services, Addis Ababa, Ethiopia; 9 Ministère de la Santé, Programme Oncho-Cécité-MTN, Conakry, République de Guinée; 10 Department of Ophthalmology, Blantyre Institute for Community Ophthalmology, College of Medicine, Blantyre, Malawi, Malawi; 11 Disease Prevention and Control Directorate, Federal Ministry of Health, Addis Ababa, Ethiopia; 12 Sightsavers, Haywards Heath, United Kingdom; 13 Programme National de la Santé Oculaire et de la Lutte contre l'Onchocercose, Abidjan, Côte-d'Ivoire; 14 Programa Nacional de Oftalmologia, Maputo, Mocambique; 15 Department of Ophthalmology, University of Jos, Jos, Nigeria; 16 Sightsavers, Kaduna, Nigeria; 17 Direction de Lutte contre la Maladie, Kinshasa, Ministere de la Santé Publique, Republique Democratique du Congo; 18 Department of Public Health, Federal Ministry of Health, Abuja, Nigeria; 19 Eye Department, Ministry of Health and Medical Services, Honiara, Solomon Islands; 20 National Ophthalmology Center, Ministry of Health, Vientiane, Lao People’s Democratic Republic; 21 World Health Organization, Port Vila, Vanuatu; 22 Clinical Research Department, London School of Hygiene & Tropical Medicine, London, United Kingdom; 23 Department of Control of Neglected Tropical Diseases, World Health Organization, Geneva, Switzerland; RTI International, UNITED REPUBLIC OF TANZANIA

## Abstract

**Background:**

Facial cleanliness and sanitation are postulated to reduce trachoma transmission, but there are no previous data on community-level herd protection thresholds. We characterize associations between active trachoma, access to improved sanitation facilities, and access to improved water sources for the purpose of face washing, with the aim of estimating community-level or herd protection thresholds.

**Methods and findings:**

We used cluster-sampled Global Trachoma Mapping Project data on 884,850 children aged 1–9 years from 354,990 households in 13 countries. We employed multivariable mixed-effects modified Poisson regression models to assess the relationships between water and sanitation coverage and trachomatous inflammation—follicular (TF). We observed lower TF prevalence among those with household-level access to improved sanitation (prevalence ratio, PR = 0.87; 95%CI: 0.83–0.91), and household-level access to an improved washing water source in the residence/yard (PR = 0.81; 95%CI: 0.75–0.88). Controlling for household-level water and latrine access, we found evidence of community-level protection against TF for children living in communities with high sanitation coverage (PR_80–90%_ = 0.87; 95%CI: 0.73–1.02; PR_90–100%_ = 0.76; 95%CI: 0.67–0.85). Community sanitation coverage levels greater than 80% were associated with herd protection against TF (PR = 0.77; 95%CI: 0.62–0.97)—that is, lower TF in individuals whose households lacked individual sanitation but who lived in communities with high sanitation coverage. For community-level water coverage, there was no apparent threshold, although we observed lower TF among several of the higher deciles of community-level water coverage.

**Conclusions:**

Our study provides insights into the community water and sanitation coverage levels that might be required to best control trachoma. Our results suggest access to adequate water and sanitation can be important components in working towards the 2020 target of eliminating trachoma as a public health problem.

## Author summary

Trachoma is the leading infectious cause of blindness. Previous association studies have primarily assessed household-level exposures, ignoring potential community-level protection from water and sanitation coverage in neighboring houses. There is biological plausibility that increased community-level coverage of facial cleanliness and/or sanitation could reduce trachoma transmission, even to non-face washers or to those without access to sanitation. Our study investigates relationships between active trachoma and community-level coverage of sanitation and water, is novel in concept and unprecedented in scale, including data from trachoma-endemic areas of 13 countries. Our findings support the plausibility of community-level or herd protection from trachoma with increasing water and sanitation coverage. We also observed lower TF prevalence among those with household-level access to sanitation and water. Our results suggest access to adequate water and sanitation can be important components in working towards the 2020 target of eliminating trachoma as a public health problem.

## Introduction

Trachoma is the leading infectious cause of blindness [1]. An estimated 450,000 people suffer from trachoma-related blindness with another 1.4 million suffering from trachoma-related moderate to severe visual impairment globally [2]. Trachoma is a public health problem in 42 countries, where 200 million people live in endemic areas [3]. Repeated conjunctival *Chlamydia trachomatis* infection, transmitted via synanthropic flies or person-to-person contact, causes scarring, and eventually (in some people) makes the eyelashes curl inwards, scraping the cornea, causing physical pain and leading to impaired vision [4]. Repeated infections are also associated with broader consequences such as poverty and social exclusion.[5, 6] WHO recommends the SAFE strategy to eliminate trachoma [7]: Surgery to reposition in-turned eyelashes; Antibiotics, given via annual mass treatment; Facial cleanliness to reduce transmission; and Environmental improvement, particularly access to water and sanitation, which facilitates facial cleanliness and reduces fly-breeding sites, respectively [8]. F and E are primary preventive interventions, and A and S are secondary and tertiary preventive interventions, respectively. S and A have a solid evidence base [9, 10]. For F and E, observational studies support implementation through associations between trachoma, poor sanitation and inadequate facial cleanliness, though evidence from individual intervention studies has been mixed [11].

Previous association studies have primarily assessed household-level exposures, ignoring potential community-level protection from water and sanitation coverage in neighboring houses. Sanitation is a public good, and ignoring community-level coverage may lead to underestimates of the importance of these exposures [12]. There is biological plausibility that increased community-level coverage of facial cleanliness and/or sanitation could reduce trachoma transmission, even to non-face washers or to those without access to sanitation. There is evidence to suggest that sanitation confers community-level or herd protection on some other health outcomes, such as anthropometric nutritional outcomes [12–15], diarrhea [16, 17], and infant mortality [18]. A cluster-randomized trial in Ethiopia demonstrated a protection effect against ocular *C*. *trachomatis* infection in untreated older individuals when 1–10-year-olds were given antibiotics [19]. Some community-based studies have assessed the impact of sanitation [20] or face washing promotion [21] on trachoma, but only one study that we know of has assessed if sanitation confers herd/community-level protection on trachoma.[22] In this study, Oswald *et al*. found that higher community coverage levels of sanitation were associated with lower prevalence of active trachoma in Ethiopia. We aren’t aware of any studies assessing impacts of community-level facewashing on trachoma.

In this study, we used data from 13 countries that participated in the Global Trachoma Mapping Project (GTMP) [23, 24]. We explore community-level coverage thresholds of sanitation and of water for face washing, seeking evidence for community-level protection (i.e., protection due to high community-level coverage) and for herd protection (i.e., protection due to high community coverage that specifically benefits those without individual access to latrines or water). We hypothesized we would observe indications of community or herd protection among individuals living in communities with high coverage of sanitation or high coverage of water for face washing.

## Methods

### Study context

All 29 GTMP-participating countries were eligible for inclusion in this study [23, 24]. All countries were sent emails requesting participation, and those interested signed agreements to collaborate and share data. Of the Ministries of Health that responded, 13 countries had adequate WASH data and were included in the final dataset. The GTMP was generally administered at national level of participating countries, and consisted of common data collection methodologies implemented by uniformly trained fieldworkers who had been certified in diagnosing TF; this training and certification is discussed in great detail elsewhere [25].

### Study population

**Fig 1:**
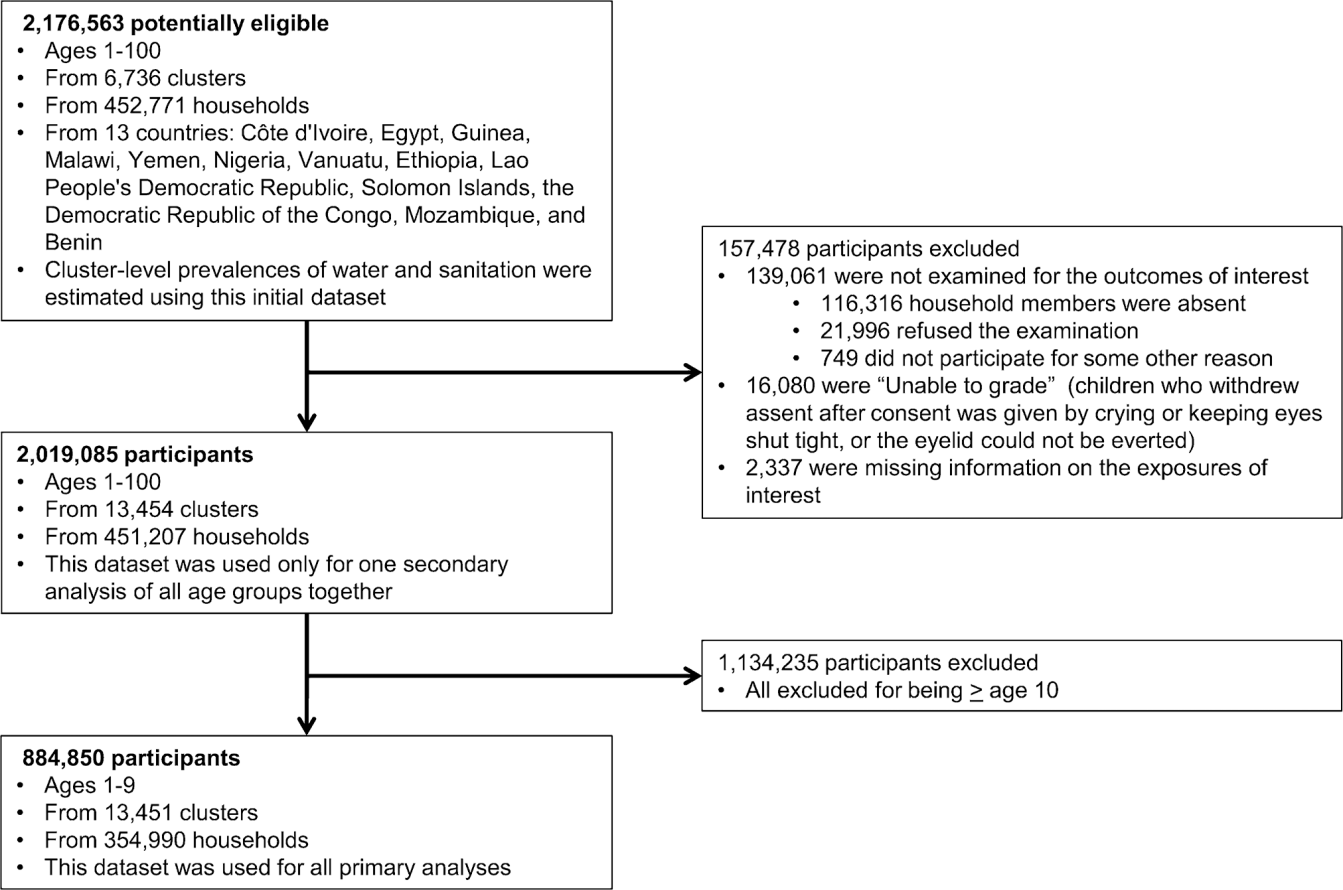
Study flow diagram.

Our dataset consisted of 2,176,563 residents from 13 countries: Côte d'Ivoire, Egypt, Guinea, Malawi, Yemen, Nigeria, Vanuatu, Ethiopia, Lao People's Democratic Republic, Solomon Islands, Democratic Republic of the Congo, Mozambique, and Benin (Fig 1). We excluded 157,478 individuals: 116,316 were absent, 21,996 refused participation, 749 had mental or physical impairments that prevented participation, 16,080 could not be examined (because, e.g., they kept eyes shut tight), and 2,337 had missing data on water and sanitation exposures. In analyses assessing all ages, therefore, we used data from 2,019,085 participants from 451,207 households in 13,454 clusters. However, our primary focus was on 1–9-year-olds, the standard active trachoma indicator group [8]. Of retained participants, 1,134,235 were aged ≥10 years, so the primary outcomes dataset included 884,850 1–9-year-olds from 354,990 households in 13,451 clusters.

### Data collection and follow-up timeline

Data were collected between December 2012 and January 2016 by health ministry staff who had been trained and certified by the GTMP [23]. Households were sampled using two-stage or multi-stage cluster sampling, employing, as far as was practical, equal-probability sampling approaches [25]. In each selected household, information was collected from the household head on the type of water source used in the dry season for drinking water, the time taken to collect water from that source, the type of water source used in the dry season for washing faces, the time taken to collect water from that source, and the usual place of defecation for household adults. Teams then visited the household’s latrine or toilet, and recorded whether hand-washing facilities, hand-washing water, and soap were present. Survey forms are provided as supplementary materials (S1 and S2 Texts). Both eyes of all household members aged ≥1-year were examined for trachoma. All data were entered directly into smartphones via a custom-built Android app [25]. Other aspects of data collection, including country-specific details, are described elsewhere [3, 23, 25–40].

### Outcome

Presence or absence of trachomatous inflammation—follicular (TF) and trachomatous inflammation—intense (TI) were each assessed in the right and left eyes. Presence of either sign is diagnostic of “active trachoma” using WHO’s simplified grading scheme [41]. Our primary outcome was TF in (either or both eyes of) 1–9-year-olds. Our primary outcome, assessing TF only, was chosen because tests for TF had a higher positive predictive value than for TI, and inter-grader agreement exercises for trainee graders used TF. Furthermore, we used TF, rather than TI (or TF and/or TI), because it is the index recommended by WHO for determining the needs for the A, F, and E interventions against trachoma. We performed a sensitivity analysis with the outcome as TF *and/or* TI in either the right or left eye or both. We also performed a secondary analysis to assess the association between TF in all-ages (rather than just 1–9-year-olds) and our exposure variables.

### Predictors

Our household-level exposures of interest were: binary household-level access to improved sanitation (i.e., improved *vs*. not), and binary household-level access to an improved source of water for face-washing located in the residence/yard (i.e., improved and on site *vs*. not). Each households’ sanitation facilities were observed by an enumerator who recorded the latrine/toilet type. We categorized sanitation facilities and water sources as improved or unimproved using WHO-UNICEF Joint Monitoring Program (JMP) for Water Supply and Sanitation definitions [42]. The main type of water source used for face washing during the dry season was reported to the enumerator by the head of household. We first categorized the water source as either improved or unimproved, as per the JMP definition [42]. The head of household also reported the distance to this water source, and because water use behaviors probably depend on distance to source [43], we constrained the definition of “improved” water sources to those located in the residence/yard. For brevity, throughout this paper we use “household sanitation” to mean binary household-level access to improved sanitation, and the term “household water” to mean binary household-level access to an improved face washing water source in the residence/yard.

Our two community-level exposure variables of interest were: the proportion of sampled households in the cluster with improved sanitation, and the proportion of sampled households in the cluster with an improved face washing water source in the residence/yard. For each individual, we estimated the surrounding prevalence of water/sanitation by aggregating household-level water/sanitation variables across the cluster, excluding that individual’s household. Including only neighboring households better represents the indirect exposure we wished to measure, and avoids forced correlation between household-level and cluster-level variables. These continuous cluster-level washing water/sanitation variables were later categorized with cut-points at each 10^th^ percentile of coverage. Many GTMP-supported surveys used compact segment sampling, and our cluster-level coverage estimates in those cases are representative of true cluster-level prevalences. For brevity, we use herein the terms “sanitation coverage,” and “water coverage” to describe our two community-level exposures of interest.

We incorporated the following potential confounders in the models: participant’s age and sex, cluster-level TF prevalence, and country (indicator variables included for each country). With infectious disease outcomes it is common to control for baseline or cluster-level prevalence of the outcome, as this variable may affect the probability of transmission to unaffected individuals [44, 45]. We employed interaction terms to jointly characterize impact of community-level and household-level water/sanitation.

### Code availability

Our analysis code is available by request to the corresponding author.

### Data analysis

All analyses were carried out in STATA, version 14 (StataCorp, College Station, TX). To explore relationships between continuous water coverage and TF and between continuous sanitation coverage and TF, we used a log-linear binomial model and a simple linear spline with knots at each centile of water/sanitation coverage. We experimented with different placement of knots, and placed knots at each centile because it allowed us to look for deviations from nonlinearity while maintaining adequate sample size and precision within groups.

For the fully adjusted models, we employed mixed-effects modified Poisson regression; log-linear binomial models were first attempted but did not converge [46]. We produced adjusted prevalence ratios (PRs) comparing various sanitation and water exposures and presence or absence of TF. Our first model assessed the relationship between TF and household- and community-level water variables, and household- and community-level sanitation variables. For community-level variables, we used indicator variables to denote water and sanitation coverage deciles within each cluster. We also used this model to explore the “total effect” of the community- and household-level variables together: we compared the TF prevalence for individuals living in the highest coverage decile who also had household latrines to the TF prevalence of individuals living in the lowest coverage decile who did not have household latrines. This fully adjusted model resembled the form:

$$\begin{array}{l} log(\mu_{ij})=\alpha +\beta_{0}HH\;{sanitation}_{ij}+\beta_{1}HH\;{water}_{ij}+\sum_{p=2}^{P=10}\beta_{p}sanitation\;{coverage}_{j}\\ \;+\sum_{q=2}^{Q=10}\beta_{q}water\;{coverage}_{j}+\sum_{r=1}^{R}\gamma_{r}\;confounde{rs}_{ij}+u_{j} \end{array}$$

where *μ*_*ij*_ represents the expected probability of the outcome in the i^th^ participant from the j^th^ cluster; *β* represents sanitation and water coefficients, and *γ* represents confounder coefficients. The subscript *p* indexes each sanitation coverage decile (omitting the reference group), *q* indexes each washing water coverage decile (omitting the reference group), and *r* indexes each confounder variable. A random intercept, *u*_j,_ is included to account for clustering within the *j*^th^ community.

To assess for linearity between water/sanitation coverage and TF, we used a similar model, but instead of including ten separate indicator variables, we included a ten-level ordinal variable.

To jointly characterize the interaction between community- and household-level water/sanitation variables, we created a second model. This interaction model allowed us to explore a possible “indirect effect” [15] of community-level coverage among those lacking household-level access. The fully adjusted interaction model resembled the form:

$$\begin{array}{l} log\left(\mu_{ij}\right)=\alpha +\beta_{0}HH\;sanitation_{ij}+\beta_{1}HH\;water_{ij}+\sum_{p=2}^{P=10}\beta_{p}sanitation\;coverage{\;}_{j}\\ \;+\sum_{q=2}^{Q=10}\beta_{q}water\;coverage_{j}+\sum_{r=1}^{R}\gamma_{r}\;confounders_{ij}\;\\ \;+\sum_{p=2}^{P=10}\delta_{p}\;sanitation\;coverage_{j}\times HH\;sanitation_{ij}\\ \;+\sum_{q=2}^{Q=10}\delta_{p}\;water\;coverage_{j}\times \mathrm{HH}\;water_{ij}+u_{j} \end{array}$$

Interaction coefficients in the above are represented by *δ*. The “Sanitation coverage × HH sanitation” term captures interactions between the *p*^th^ sanitation coverage decile and the household sanitation variable. Similarly, the “Water coverage_j_ × HH water_ij_” terms capture interactions between the *q*^th^ washing water coverage decile and the household water variable.

We performed a sensitivity analysis to assess the association between any sanitation use (rather than improved sanitation) and TF. We also performed a sensitivity analysis to assess the association between any washing water located in the residence/yard (rather than improved and located in the residence/yard) and TF. Finally, we performed a sensitivity analysis to assess the association between having a washing water source within 30 minutes compared to ≥30 minutes and TF. For each of these sensitivity analyses, the household sanitation and water variables were aggregated to create a community-level variable (analogous to our creation of coverage variables for the primary analyses). Each model was similar to the first model described above, substituting the new variable of interest.

Our main analyses used data from all 13 countries with the goal to improve generalizability. However, we also did some additional analyses on specific sub-populations to further asses internal validity. *Musca sorbens* is not known in Vanuatu, Lao, or the Solomon Islands, so we performed a sensitivity analysis to assess our sanitation findings, without including these three countries. Another reason for using data from all 13 countries, is that the models require lots of observations. Nigeria contributed enough data (and had enough variability in their data) to run a model specific to Nigeria only, and we present a sensitivity analysis using this country only.

### Ethical approval

It was determined by the Emory IRB that no IRB review was required for our secondary analyses on de-identified data (IRB00091226).

## Results

### Descriptive statistics for TF, sanitation, and water

The final dataset consisted of 884,850 1–9-year-olds from 354,990 households from 13 countries (Table 1). Of these 884,850 1–9-year-olds, 8.2% (SE = 0.1%) had TF. TF prevalence was lower when including participants of all ages (prevalence = 4.4%; SE = 0.1%; S1 Table). The intra-cluster correlation coefficient for TF was 0.54. Of 354,990 included households, 18.1% (SE = 0.3%) had household sanitation, and 11.5% (SE = 0.2%) had household water (Table 1). Prevalences of TF, household sanitation, and household water varied across countries.

**Table 1:** Descriptive results for trachomatous inflammation—Follicular, improved face-washing water source in the residence/yard, and improved sanitation prevalences for children aged 1–9 years.

	Participant-level (1–9-year-olds)		Household-level			Cluster-level
	N participants	% with TF^a^ (%SE)^b^	N households	% with improved san. (%SE)^b^^,^^c^	% with improved water (%SE)^b^^,^^d^	N clusters
Total	884,850	8.2 (0.1)	354,990	18.1 (0.3)	11.5 (0.2)	13,451
By country						
Côte d'Ivoire	17,704	10.3 (0.6)	6,907	0.5 (0.1)	9.2 (1.5)	257
Egypt	3,682	17.3 (1.7)	2,065	99.3 (0.7)	99.7 (0.1)	100
Guinea	19,660	4.3 (0.3)	7,677	8.8 (1.2)	8.8 (0.9)	219
Malawi	34,397	6.4 (0.3)	17,116	5.3 (0.5)	4.7 (0.6)	677
Yemen	56,064	3.1 (0.2)	24,189	71.1 (1.3)	50.3 (1.6)	965
Nigeria	341,076	3 (0.1)	116,907	18.1 (0.4)	11.8 (0.3)	3,994
Vanuatu	868	15 (2.5)	532	47.6 (5.5)	34.6 (5.9)	42
Ethiopia	174,628	22.6 (0.4)	84,585	5.6 (0.2)	1.9 (0.2)	4,324
Lao People's Democratic Republic	21,511	1 (0.1)	12,477	57.7 (2.3)	46.6 (2.6)	320
Solomon Islands	3,005	19.5 (1.6)	1,686	11.3 (2)	23.1 (3)	82
Democratic Republic of the Congo	83,061	9.3 (0.3)	30,984	8.8 (0.7)	2.1 (0.3)	1,162
Mozambique	83,188	4.3 (0.2)	42,511	16.1 (0.8)	3.9 (0.4)	1,048
Benin	46,006	7.7 (0.8)	7,354	4.3 (0.8)	5.3 (0.7)	261

^a^ Trachomatous inflammation—follicular in either or both eyes.

^b^ We accounted for clustering in the standard error estimates.

^c^Improved sanitation, as defined by the JMP [42].

^d^ Improved water, as defined by the JMP [42], but with the additional constraint that the water source had to be located in the residence/yard.

### Univariable analyses

**Fig 2:**
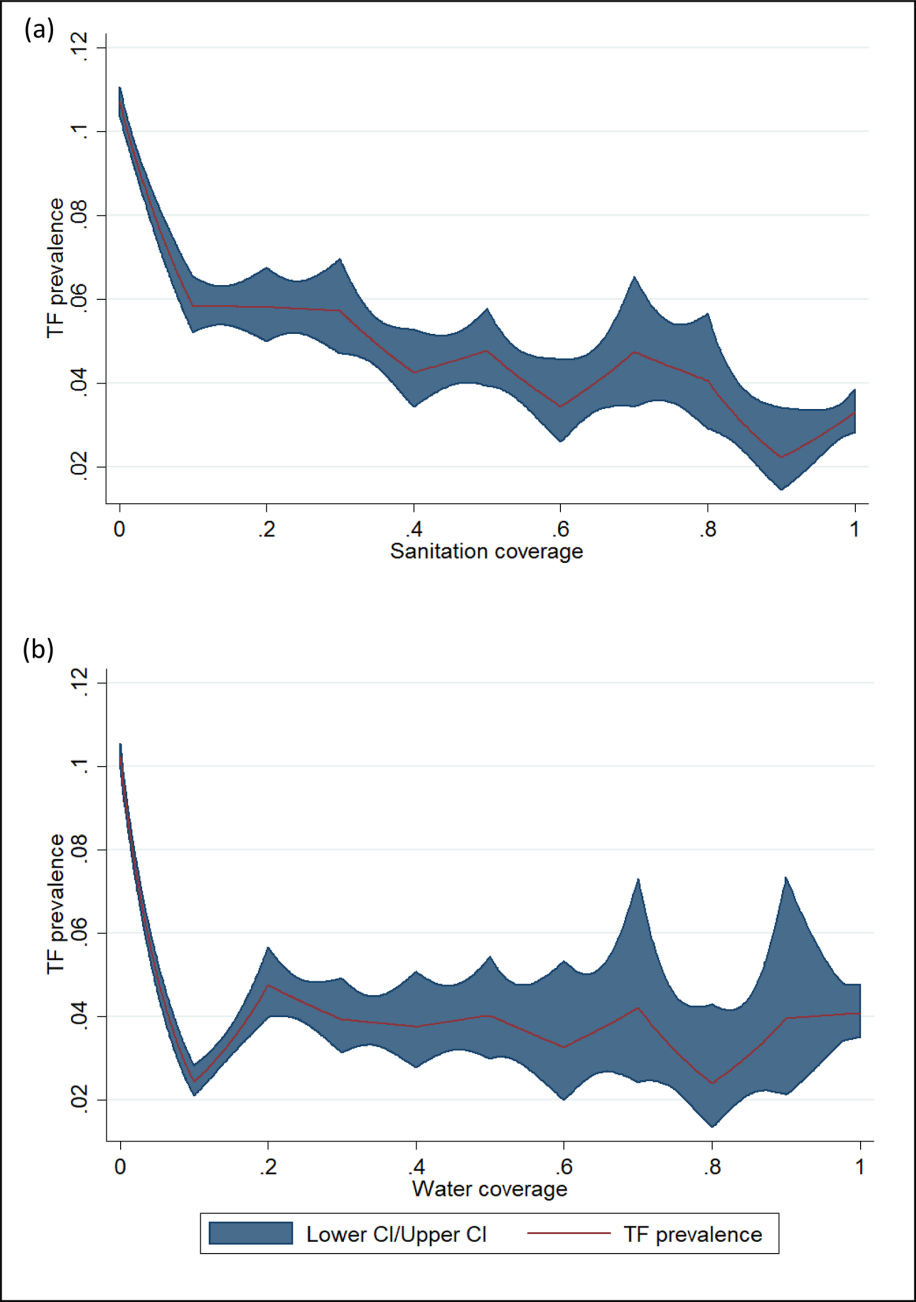
Unadjusted relationship between sanitation coverage (a) or water coverage (b) and prevalence of trachomatous inflammation—follicular (TF) among children aged 1–9 years.

Unadjusted analyses showed that communities in the lowest water and sanitation coverage decile had the highest TF prevalence (Fig 2). As sanitation coverage increased from 0% to 100%, the TF prevalence generally decreased (Fig 2 (A)). As water coverage increased from 0% to 10%, there was a steep decrease in TF prevalence, after which the TF prevalence remained relatively flat in the 20–100% coverage range (Fig 2 (B)). The high TF prevalence in the first deciles of water and sanitation coverage were heavily driven by data from Ethiopia, which contributed 174,628 1–9-year-olds with high TF prevalence (22.6%), and very low household water (1.9%) and sanitation (5.6%).

### Multivariable analyses

**Fig 3:**
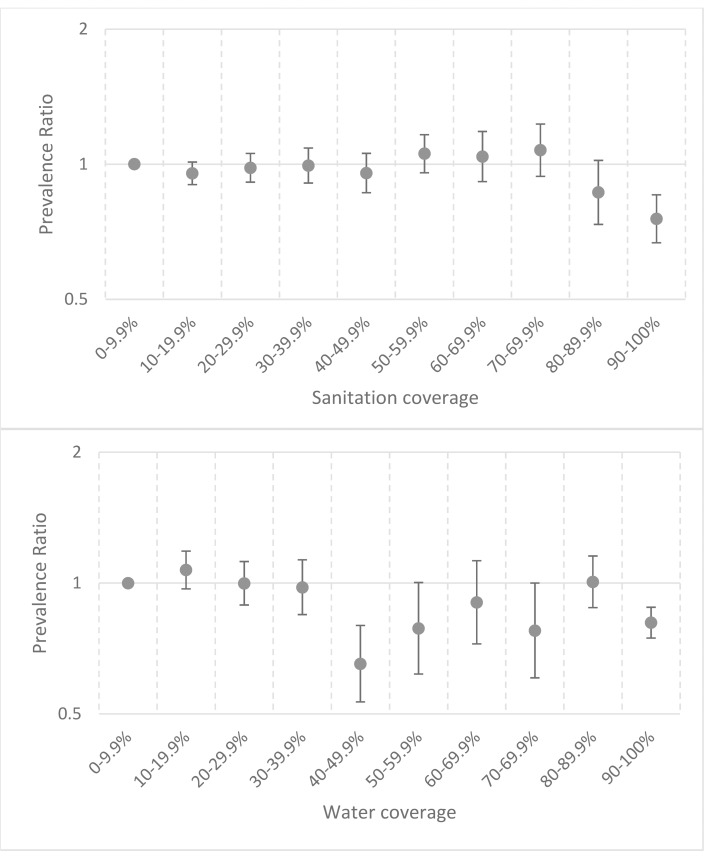
Results from multivariable model showing the association between community-level sanitation and water coverage on trachomatous inflammation—Follicular prevalence among children aged 1–9 years.

Our first model assessed the relationship between TF and household- and community-level water variables, and household- and community-level sanitation variables. TF prevalence was lower among those with household sanitation (prevalence ratio [PR] = 0.87; 95% CI: 0.83, 0.91; Table 2), compared to those without. A lower TF prevalence was also found among those with household water (PR = 0.81; 95% CI: 0.75, 0.88) compared to those without. When considering community-level sanitation coverage, we observed lower levels of TF for participants living in communities with at least 90% sanitation coverage (PR_90–100%_ = 0.76; 95% CI: 0.67, 0.85) compared to those living in communities with 0–10% coverage. We also observed lower TF levels, although marginally insignificant (*p =* 0.09), for participants living in communities with 80–90% latrine coverage (PR_80–90%_ = 0.87; 95% CI: 0.73, 1.02). As for washing water coverage, several of the estimates comparing higher coverage deciles to the lowest coverage decile had lower prevalences of TF (Table 2; Fig 3).

**Table 2:** Multivariable model showing the household-level and community-level associations between sanitation, water and trachomatous inflammation—Follicular (TF) among children aged 1–9 years.

	Prevalence ratio (95% CI)^a^	*p-value*	*p-trend*
*Sanitation variables*			
Household sanitation (yes vs. no)^b^	0.87 (0.83, 0.91)	<0.01**	n/a
Sanitation coverage (%)^b^^,^ ^d^			<0.01**
0–9.9%	ref		
10–19.9%	0.95 (0.90, 1.01)	0.12	
20–29.9%	0.98 (0.91, 1.06)	0.63	
30–39.9%	0.99 (0.91, 1.09)	0.89	
40–49.9%	0.96 (0.86, 1.06)	0.38	
50–59.9%	1.06 (0.96, 1.16)	0.28	
60–69.9%	1.04 (0.91, 1.18)	0.55	
70–69.9%	1.07 (0.94, 1.23)	0.29	
80–89.9%	0.87 (0.73, 1.02)	0.09*	
90–100%	0.76 (0.67, 0.85)	<0.01**	
*Water variables*			
Household water (yes vs. no)^c^	0.81 (0.75, 0.88)	<0.01**	n/a
Water coverage (%)^c^^,^ ^d^			0.04**
0–9.9%	ref		
10–19.9%	1.07 (0.97, 1.18)	0.18	
20–29.9%	1.00 (0.89, 1.12)	0.99	
30–39.9%	0.98 (0.85, 1.13)	0.77	
40–49.9%	0.65 (0.53, 0.80)	<0.01**	
50–59.9%	0.79 (0.62, 1.00)	0.05*	
60–69.9%	0.90 (0.72, 1.13)	0.36	
70–69.9%	0.78 (0.61, 1.00)	0.05*	
80–89.9%	1.01 (0.88, 1.15)	0.93	
90–100%	0.81 (0.75, 0.88)	<0.01**	
Other included confounders not shown^a^	.^a^		

* = significant at 0.1 level.

** = significant at 0.05 level.

^a^ The model controlled for all variables shown in the table and additionally controlled for country, prevalence of TF in the cluster, participant’s age, and participant’s sex; it included a random effect to account for clustering.

^b^Improved sanitation, as defined by the JMP [42].

^c^ Improved water, as defined by the JMP [42], but with an additional constraint that the water source had to be located in the residence/yard.

^d^ These community-level results are shown graphically in Fig 3.

To assess for linearity between water/sanitation coverage and TF, we used a similar model, but instead of including ten separate indicator variables, we included a ten-level ordinal variable. There was a linear trend between sanitation coverage and TF (*p*-trend = 0.008; Table 2); however, this trend was driven largely by decreases in TF prevalence only at coverage >80% (Fig 3). There was a linear trend (*p*-trend = 0.038; Table 2) between water coverage and TF; however, the graphical representation of this relationship is more V-shaped than linear (Fig 3).

**Fig 4:**
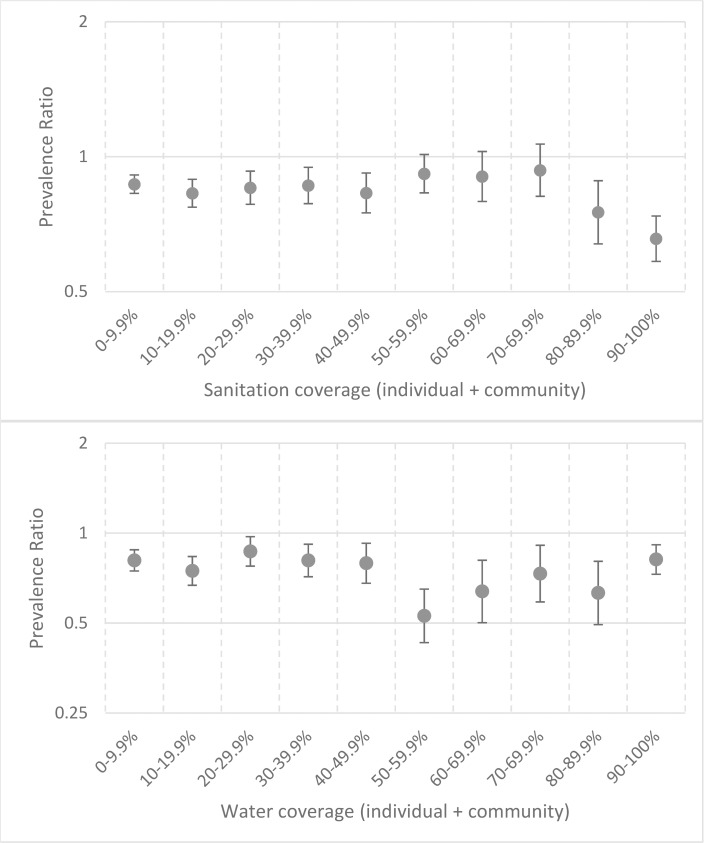
Association between trachomatous inflammation—Follicular in children aged 1–9 years and both household and community associations combined together (i.e. the “total effect”). The reference group is participants without household washing water/sanitation living in the lowest coverage decile.

We also use parameters from the first model to characterize the “total effect” of the community- and household-level variables together. The PRs due to having sanitation both at home and across the community compared to not having sanitation in either place were highly significant (PR_80–90%+home_ = 0.75; 95% CI: 0.64, 0.88; PR_90–100%+home_ = 0.65; 95% CI: 0.58, 0.74; Fig 4). The PRs contrasting the “total effect” due to having water both at home and across the community compared to not having water in either place were all significant (Fig 4).

**Fig 5:**
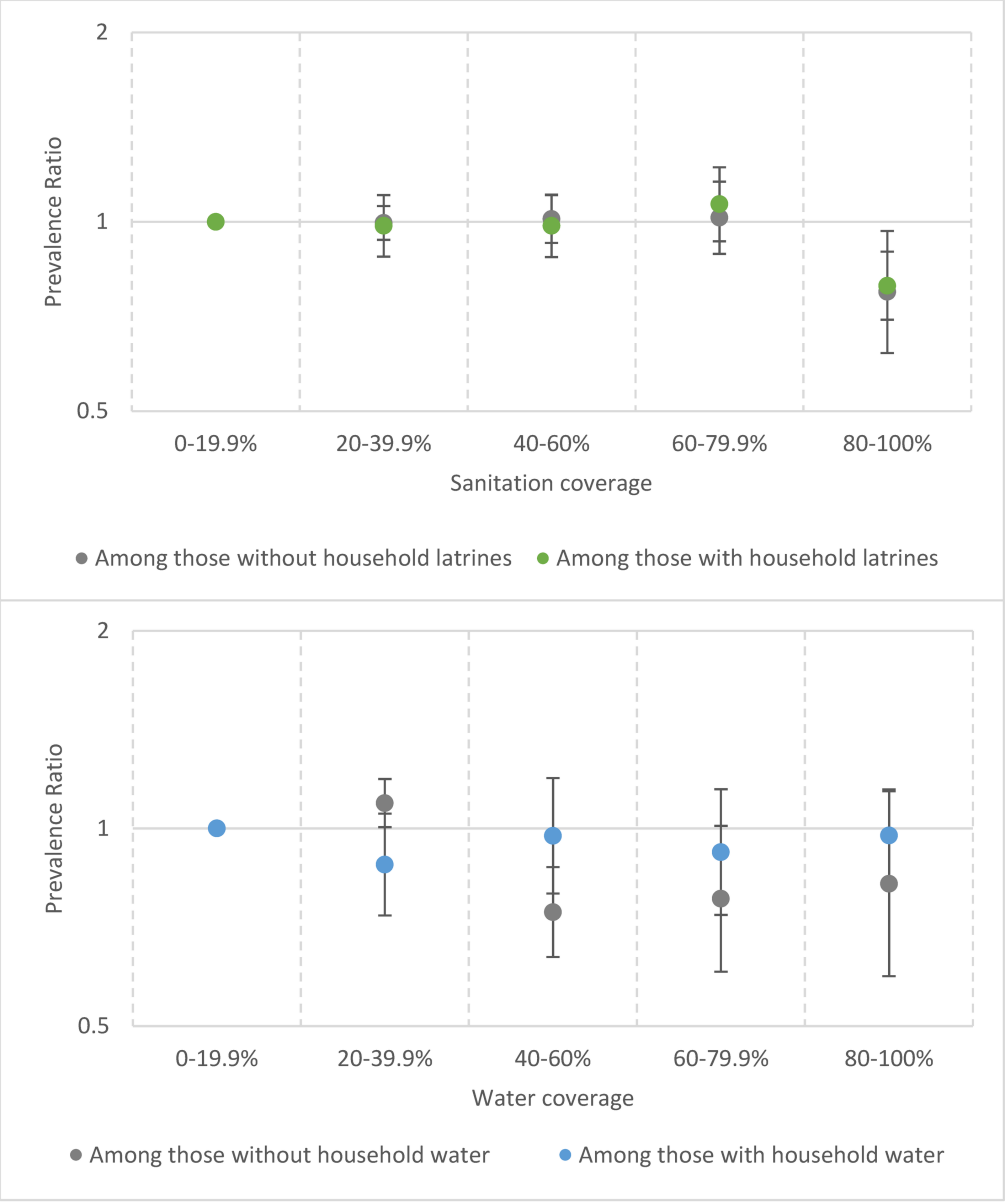
Results from multivariable interaction model showing the association between community-level sanitation coverage and water coverage on trachomatous inflammation—Follicular prevalence, stratified by household access to sanitation or water among children aged 1–9 years.

To jointly characterize the interaction between community- and household-level water/sanitation variables, we used the model with interaction terms. Our results indicated evidence for “herd protection” at sanitation coverage ≥80% (PR = 0.77; 95% CI: 0.62, 0.97; Fig 5). There was no clear relationship between water coverage and TF (Fig 5). Using deciles instead of quintiles with these interaction terms led to many estimates having wide confidence intervals (S1 Fig), so our preferred analysis was that which used quintiles (Fig 5).

### Sensitivity analyses

Our sensitivity analysis to assess the association between TF and our exposure variables in *all-ages* (rather than just 1–9-year-olds) showed a nearly identical relationship between water and sanitation coverages and TF (S2 Table; S2 Fig).

Our sensitivity analysis to assess the association between *any sanitation use* (rather than improved sanitation) and TF showed no evidence of lower TF with increasing sanitation coverage (S3 Table; S3 Fig). The sensitivity analysis to assess the association between any washing water located in the residence/yard (rather than improved and located in the residence/yard) and TF showed evidence of lower TF in the upper two water coverage deciles (S4 Table; S4 Fig). The sensitivity analysis to assess the association between having a washing water source within 30 minutes compared to ≥30 minutes and TF showed no evidence of lower TF (and perhaps an increase) with increasing community-level water coverage (S5 Table; S5 Fig). The sensitivity analysis with the outcome as TF and/or TI produced results that were nearly identical to those from the main analysis assessing TF only (S6 Table; S6 Fig). In the sensitivity analysis in which we dropped those countries where *Musca sorbens* is not known, the sanitation results were very similar to those results from all countries (S7 Fig). Finally, in the sensitivity analysis where we analyzed the Nigeria data alone, we observed associations that indicate community-level protection against TF due to high coverage of sanitation, but also protection due to high coverage of water (S8 Fig).

## Discussion

Our study investigates relationships between active trachoma and community-level coverage of sanitation and water, and is noteworthy in scale, including data from trachoma-endemic areas of 13 countries. Our findings support the plausibility of community-level protection from trachoma with increasing water and sanitation coverage. We also observed lower TF prevalence among those with household-level access to sanitation and water.

Our study had some limitations. Communities with widely different water/sanitation coverage probably have different socio-economic status (SES) [47]. Although trachoma has previously been associated with poverty [47, 48], foundations for the routes of ocular *C*. *trachomatis* transmission are probably buttressed more by the environmental conditions (such as poor sanitation and hygiene) that are byproducts of lower SES, than by low SES itself. Another limitation is that our assessment of access to water and sanitation may not correspond with water, sanitation, and hygiene behaviors, such as sanitation use and clean face behaviors. For example, the existence of a latrine does not necessarily mean that it is used by everyone, all the time. Finally, our community-level variables were measured in clusters which varied in size and population density, and may not actually correlate with intensity of transmission at community level.

Pooled estimates from a recent systematic reviews and meta-analyses assessing water, sanitation and hygiene for trachoma [11, 49] are similar in direction and magnitude to our household results. Specifically, the odds ratios from the systematic review were 0.85 for household sanitation (our PR for household sanitation was 0.87) and 0.76 for daily face washing (our PR for household water was 0.81). We only found the Oswald *et al*. study that assessed community coverage levels of sanitation and prevalence of active trachoma in Ethiopia, and both the Oswald study and our study found that sanitation coverage ≥80% was associated with lower TF prevalence. Our community-level results showed the lowest trachoma prevalence in communities where sanitation coverage was ≥ 90%. We also observed evidence of protection against trachoma in communities where sanitation coverage levels were ≥ 80%, even for those without individual household latrines, implying that high sanitation coverage might confer herd protection. The “total effects’ we observed (among those with household sanitation living in the highest sanitation deciles), incorporate both the direct and indirect contributions of sanitation on TF, and were much stronger; studies not considering community-level sanitation coverage may underestimate the effect of sanitation [12] on trachoma. The results from other trachoma and sanitation or water studies in the literature have been mixed, [50–55], perhaps in part because several of these interventions did not achieve or barely achieved the very high coverage levels (e.g., 80–90%) that might be required to confer community-level protection.

Sanitation is thought to reduce the prevalence of active trachoma by minimizing fly breeding sites: the fly vector *Musca sorbens* preferentially oviposits on soil-exposed human feces. Flies are not constrained to given household environments but can circulate amongst the community, making community-level/indirect effects plausible. Given that exposure of feces is key, our sensitivity analysis assessing coverage of any type of sanitation is interesting: it showed no signs of community-level protection. Unimproved sanitation facilities, such as open pits, may not adequately prevent flies from laying their eggs. Similarly, our sensitivity analysis assessing coverage of water from sources up to 30 minutes away showed no signs of community-level protection from higher coverage, whereas it is usually hypothesized that water farther away than this is less likely to be used for face washing than on-site water.

### Conclusions

Our results are congruent with the belief that water, sanitation and hygiene are important for accelerating efforts towards global trachoma elimination as a public health problem [3, 56]. Our findings are also in support of Sustainable Development Goal 6, which calls for availability and sustainable management of water and sanitation for all [57]. We provide some evidence of the importance of reaching high water and sanitation coverage levels in order to confer health benefits. While we observed the lowest TF prevalences among people living in the highest sanitation coverage deciles, actually attaining these high sanitation coverage levels may take significant effort, as non-adopters tend to be of lower SES, more marginalized, less educated, and often living in more difficult-to-reach locations [58, 59]. A systematic review assessing the impact of sanitation interventions suggested that only 7 of 27 interventions would achieve sanitation coverage of >80% [60], the level that our data suggests might be required to confer community-level or herd protection against trachoma. Achieving higher sanitation among lower SES groups might be particularly beneficial, in that trachoma is more likely to affect such populations [47, 48]. Our findings indicate that even if communities are initially unable to attain the high community-wide sanitation prevalences that might be required to attain community-level or herd protection, there may still be direct benefits of individual households having access to both sanitation and washing water.

## Supporting information

S1 Checklist STROBE checklist. (DOC)

S1 Fig Results from interaction model showing the association between community-level sanitation on trachomatous inflammation—Follicular prevalence, stratified by household-level access to sanitation. (TIF)

S2 Fig Results from multivariable model showing the association between community-level sanitation and water coverage on trachomatous inflammation—Follicular prevalence among all ages (also see S1 Table). (TIF)

S3 Fig Results from sensitivity analysis showing the association between community-level use of any type of latrine on trachomatous inflammation—follicular prevalence (also see S2 Table). (TIF)

S4 Fig Results from sensitivity analysis showing the association between community-level coverage of washing water in the residence/yard on trachomatous inflammation—Follicular prevalence (also see S3 Table). (TIF)

S5 Fig Results from sensitivity analysis showing the association between community-level coverage of washing water within 30 minutes on trachomatous inflammation—Follicular prevalence (also see S4 Table). (TIF)

S6 Fig Results from multivariable model showing the association between community-level sanitation and water coverage on trachomatous inflammation—Follicular prevalence and/or trachomatous inflammation—intense (TI) among (also see S5 Table). (TIF)

S7 Fig Results from multivariable model showing the association between community-level sanitation and trachomatous inflammation—Follicular prevalence dropping countries where Musca sorbens is not known (i.e., dropping Vanuatu, Lao, or the Solomon Islands). (TIF)

S8 Fig Results from multivariable model showing the association between community-level sanitation and water coverage on trachomatous inflammation—Follicular prevalence among Nigeria only. (TIF)

S1 Table Descriptive results for trachomatous inflammation—Follicular, improved face-washing water source in the residence/yard, and improved sanitation prevalences for all ages. (DOCX)

S2 Table Multivariable model showing the household-level and community-level associations between improved sanitation, water and trachomatous inflammation—Follicular (TF) among all ages. (DOCX)

S3 Table Multivariable model showing the household-level and community-level associations between any type of latrine (*vs*. no latrine/open defecation), washing water and trachomatous inflammation—Follicular (TF) among ages 1–9. (DOCX)

S4 Table Multivariable model showing the household-level and community-level associations between sanitation, washing water in the residence/yard (*vs*. not) and trachomatous inflammation—Follicular (TF) among ages 1–9. (DOCX)

S5 Table Multivariable model showing the household-level and community-level associations between improved sanitation, washing water within 30 minutes (*vs*. not) and trachomatous inflammation—Follicular (TF) among all ages 1–9. (DOCX)

S6 Table Multivariable model showing the household-level and community-level associations between improved sanitation, washing water and trachomatous inflammation—Follicular (TF) and/or trachomatous inflammation—Intense (TI) among ages 1–9. (DOCX)

S1 Text Resident survey. (PDF)

S2 Text Household survey. (PDF)

S3 Text List of contacts for data access. (PDF)

## References

[1] World Health Organization (2017). "WHO Alliance for the Global Elimination of Trachoma by 2020: progress report on elimination of trachoma, 2014–2016." Wkly Epidemiol Rec 92(26): 359–368. 28664685

[2] Bourne RR, Stevens GA, White RA, Smith JL, Flaxman SR, Price H, et al Causes of vision loss worldwide, 1990–2010: a systematic analysis. Lancet Glob Health. 2013;1(6):e339–49. doi: 10.1016/S2214-109X(13)70113-X . 25104599 10.1016/S2214-109X(13)70113-X

[3] Muhammad N, Mpyet C, Adamu MD, William A, Umar MM, Goyol M, et al Mapping Trachoma in Kaduna State, Nigeria: Results of 23 Local Government Area-Level, Population-Based Prevalence Surveys. Ophthalmic Epidemiol. 2016;23(sup1):46–54. doi: 10.1080/09286586.2016.1250918 . 27918227 10.1080/09286586.2016.1250918 PMC5706975

[4] Hu VH, Harding‐Esch EM, Burton MJ, Bailey RL, Kadimpeul J, Mabey DC. Epidemiology and control of trachoma: systematic review. Tropical Medicine & International Health. 2010;15(6):673–91. 20374566 10.1111/j.1365-3156.2010.02521.x PMC3770928

[5] Habtamu E, Wondie T, Aweke S, Tadesse Z, Zerihun M, Zewudie Z, et al The Impact of Trachomatous Trichiasis on Quality of Life: A Case Control Study. PLoS Negl Trop Dis. 2015;9(11):e0004254 Epub 2015/11/23. doi: 10.1371/journal.pntd.0004254 ; PubMed Central PMCID: PMCPMC4657886. 26598937 10.1371/journal.pntd.0004254 PMC4657886

[6] Habtamu E, Wondie T, Aweke S, Tadesse Z, Zerihun M, Zewdie Z, et al Trachoma and Relative Poverty: A Case-Control Study. PLoS Negl Trop Dis. 2015;9(11):e0004228 52. doi: 10.1371/journal.pntd.0004228 26600211 10.1371/journal.pntd.0004228 PMC4657919

[7] World Health Assembly. resolution 51.11. 1998 May 16, 1998. Report No.

[8] Solomon AW, Zondervan M, Kuper H, Buchan JC, Mabey DCW, Foster A. Trachoma control: a guide for programme managers. Geneva: World Health Organization, 2006.

[9] Burton M, Habtamu E, Ho D, Gower EW. Interventions for trachoma trichiasis. Cochrane Database Syst Rev. 2015;(11):CD004008 doi: 10.1002/14651858.CD004008.pub3 ; PubMed Central PMCID: PMCPMC4661324. 26568232 10.1002/14651858.CD004008.pub3 PMC4661324

[10] Evans JR, Solomon AW. Antibiotics for trachoma. Cochrane Database Syst Rev. 2011;(3):CD001860 doi: 10.1002/14651858.CD001860.pub3 . 21412875 10.1002/14651858.CD001860.pub3

[11] Stocks ME, Ogden S, Haddad D, Addiss DG, McGuire C, Freeman MCCP. Effect of water, sanitation, and hygiene on the prevention of trachoma: a systematic review and meta-analysis. PLoS Med. 2014;11(2):e1001605 9. doi: 10.1371/journal.pmed.1001605 24586120 10.1371/journal.pmed.1001605 PMC3934994

[12] Fuller JA, Villamor E, Cevallos W, Trostle J, Eisenberg JN. I get height with a little help from my friends: herd protection from sanitation on child growth in rural Ecuador. International journal of epidemiology. 2016 Epub 2016/03/05. doi: 10.1093/ije/dyv368 . 26936912 10.1093/ije/dyv368 PMC5841884

[13] Alderman H, Hentschel J, Sabates R. With the help of one's neighbors: externalities in the production of nutrition in Peru. Soc Sci Med. 2003;56(10):2019–31. Epub 2003/04/17. . 12697194 10.1016/s0277-9536(02)00183-1

[14] Spears D, Ghosh A, Cumming O. Open defecation and childhood stunting in India: an ecological analysis of new data from 112 districts. PloS one. 2013;8(9):e73784 Epub 2013/09/26. doi: 10.1371/journal.pone.0073784 ; PubMed Central PMCID: PMC3774764. 24066070 10.1371/journal.pone.0073784 PMC3774764

[15] Fuller JA, Eisenberg JN. Herd Protection from Drinking Water, Sanitation, and Hygiene Interventions. Am J Trop Med Hyg. 2016;95(5):1201–10. doi: 10.4269/ajtmh.15-0677 ; PubMed Central PMCID: PMCPMC5094239. 27601516 10.4269/ajtmh.15-0677 PMC5094239

[16] Barreto ML, Genser B, Strina A, Teixeira MG, Assis AM, Rego RF, et al Effect of city-wide sanitation programme on reduction in rate of childhood diarrhoea in northeast Brazil: assessment by two cohort studies. Lancet. 2007;370(9599):1622–8. doi: 10.1016/S0140-6736(07)61638-9 ; PubMed Central PMCID: PMC2212752. 17993362 10.1016/S0140-6736(07)61638-9 PMC2212752

[17] Andres LA, Briceno B, Chase C, Echenique JA. Sanitation and Externalities: Evidence from Early Childhood Health in Rural India. Washington, DC: World Bank, 2014.

[18] Geruso M, Spears D. Neighborhood Sanitation and Infant Mortality. Cambridge, MA: National Bureau of Economic Research, 2015. 10.1257/app.20150431 PMC10782420 38213507

[19] House JI, Ayele B, Porco TC, Zhou Z, Hong KC, Gebre T, et al Assessment of herd protection against trachoma due to repeated mass antibiotic distributions: a cluster-randomised trial. Lancet. 2009;373(9669):1111–8. doi: 10.1016/S0140-6736(09)60323-8 . 19329003 10.1016/S0140-6736(09)60323-8

[20] Emerson PM, Lindsay SW, Alexander N, Bah M, Dibba SM, Faal HB, et al Role of flies and provision of latrines in trachoma control: cluster-randomised controlled trial. Lancet. 2004;363(9415):1093–8. doi: 10.1016/S0140-6736(04)15891-1 . 15064026 10.1016/S0140-6736(04)15891-1

[21] West S, Munoz B, Lynch M, Kayongoya A, Chilangwa Z, Mmbaga BB, et al Impact of face-washing on trachoma in Kongwa, Tanzania. Lancet. 1995;345(8943):155–8. . 7823670 10.1016/s0140-6736(95)90167-1

[22] Oswald WE, Stewart AE, Kramer MR, Endeshaw T, Zerihun M, Melak B, et al Active trachoma and community use of sanitation, Ethiopia. Bull World Health Organ. 2017;95(4):250–60. Epub 2017/01/26. doi: 10.2471/BLT.16.177758 ; PubMed Central PMCID: PMCPMC5407250. 28479620 10.2471/BLT.16.177758 PMC5407250

[23] Solomon AW, Kurylo E. The global trachoma mapping project. Community Eye Health. 2014;27(85):18 ; PubMed Central PMCID: PMCPMC4069783. 24966461 PMC4069783

[24] Engels D. The Global Trachoma Mapping Project: A Catalyst for Progress Against Neglected Tropical Diseases. Ophthalmic Epidemiol. 2016;23(sup1):1–2. doi: 10.1080/09286586.2016.1257139 . 28030282 10.1080/09286586.2016.1257139 PMC5706979

[25] Solomon AW, Pavluck AL, Courtright P, Aboe A, Adamu L, Alemayehu W, et al The Global Trachoma Mapping Project: Methodology of a 34-Country Population-Based Study. Ophthalmic Epidemiol. 2015;22(3):214–25. doi: 10.3109/09286586.2015.1037401 ; PubMed Central PMCID: PMCPMC4687001. 26158580 10.3109/09286586.2015.1037401 PMC4687001

[26] Abashawl A, Macleod C, Riang J, Mossisa F, Dejene M, Willis R, et al Prevalence of Trachoma in Gambella Region, Ethiopia: Results of Three Population-Based Prevalence Surveys Conducted with the Global Trachoma Mapping Project. Ophthalmic Epidemiol. 2016;23(sup1):77–83. doi: 10.1080/09286586.2016.1247875 . 27918222 10.1080/09286586.2016.1247875 PMC5706976

[27] Adamu MD, Mpyet C, Muhammad N, Umar MM, Muazu H, Olamiju F, et al Prevalence of Trachoma in Niger State, North Central Nigeria: Results of 25 Population-Based Prevalence Surveys Carried Out with the Global Trachoma Mapping Project. Ophthalmic Epidemiol. 2016;23(sup1):63–9. doi: 10.1080/09286586.2016.1242757 . 27918223 10.1080/09286586.2016.1242757 PMC5706972

[28] Adamu Y, Macleod C, Adamu L, Fikru W, Kidu B, Abashawl A, et al Prevalence of Trachoma in Benishangul Gumuz Region, Ethiopia: Results of Seven Population-Based Surveys from the Global Trachoma Mapping Project. Ophthalmic Epidemiol. 2016;23(sup1):70–6. doi: 10.1080/09286586.2016.1247877 . 27918248 10.1080/09286586.2016.1247877 PMC5706978

[29] Adera TH, Macleod C, Endriyas M, Dejene M, Willis R, Chu BK, et al Prevalence of and Risk Factors for Trachoma in Southern Nations, Nationalities, and Peoples' Region, Ethiopia: Results of 40 Population-Based Prevalence Surveys Carried Out with the Global Trachoma Mapping Project. Ophthalmic Epidemiol. 2016;23(sup1):84–93. doi: 10.1080/09286586.2016.1247876 . 27918229 10.1080/09286586.2016.1247876 PMC5706981

[30] Bero B, Macleod C, Alemayehu W, Gadisa S, Abajobir A, Adamu Y, et al Prevalence of and Risk Factors for Trachoma in Oromia Regional State of Ethiopia: Results of 79 Population-Based Prevalence Surveys Conducted with the Global Trachoma Mapping Project. Ophthalmic Epidemiol. 2016;23(6):392–405. doi: 10.1080/09286586.2016.1243717 . 27820657 10.1080/09286586.2016.1243717 PMC6837860

[31] Kalua K, Chisambi A, Chinyanya D, Kamwendo Z, Masika M, Willis R, et al Completion of Baseline Trachoma Mapping in Malawi: Results of Eight Population-Based Prevalence Surveys Conducted with the Global Trachoma Mapping Project. Ophthalmic Epidemiol. 2016;23(sup1):32–8. doi: 10.1080/09286586.2016.1230224 . 27726469 10.1080/09286586.2016.1230224 PMC5706967

[32] Kalua K, Phiri M, Kumwenda I, Masika M, Pavluck AL, Willis R, et al Baseline Trachoma Mapping in Malawi with the Global Trachoma Mapping Project (GTMP). Ophthalmic Epidemiol. 2015;22(3):176–83. doi: 10.3109/09286586.2015.1035793 ; PubMed Central PMCID: PMCPMC4673584. 26158575 10.3109/09286586.2015.1035793 PMC4673584

[33] Sherief ST, Macleod C, Gigar G, Godefay H, Abraha A, Dejene M, et al The Prevalence of Trachoma in Tigray Region, Northern Ethiopia: Results of 11 Population-Based Prevalence Surveys Completed as Part of the Global Trachoma Mapping Project. Ophthalmic Epidemiol. 2016;23(sup1):94–9. doi: 10.1080/09286586.2016.1250917 . 27918232 10.1080/09286586.2016.1250917 PMC5706977

[34] Sokana O, Macleod C, Jack K, Butcher R, Marks M, Willis R, et al Mapping Trachoma in the Solomon Islands: Results of Three Baseline Population-Based Prevalence Surveys Conducted with the Global Trachoma Mapping Project. Ophthalmic Epidemiol. 2016;23(sup1):15–21. doi: 10.1080/09286586.2016.1238946 . 27937043 10.1080/09286586.2016.1238946 PMC5706973

[35] Mpyet C, Muhammad N, Adamu MD, Muazu H, Umar MM, Alada J, et al Trachoma Mapping in Gombe State, Nigeria: Results of 11 Local Government Area Surveys. Ophthalmic Epidemiol. 2016;23(6):406–11. doi: 10.1080/09286586.2016.1230633 . 27726459 10.1080/09286586.2016.1230633 PMC6839962

[36] Mpyet C, Muhammad N, Adamu MD, Muazu H, Umar MM, Goyol M, et al Prevalence of Trachoma in Katsina State, Nigeria: Results of 34 District-Level Surveys. Ophthalmic Epidemiol. 2016;23(sup1):55–62. doi: 10.1080/09286586.2016.1236975 . 27775463 10.1080/09286586.2016.1236975 PMC5751970

[37] Southisombath K, Sisalermsak S, Chansan P, Akkhavong K, Phommala S, Lewallen S, et al National Trachoma Assessment in the Lao People's Democratic Republic in 2013–2014. Ophthalmic Epidemiol. 2016;23(sup1):8–14. doi: 10.1080/09286586.2016.1236973 . 27846362 10.1080/09286586.2016.1236973 PMC5706970

[38] Mpyet C, Muhammad N, Adamu MD, Muazu H, Umar MM, Abdull M, et al Prevalence of Trachoma in Bauchi State, Nigeria: Results of 20 Local Government Area-Level Surveys. Ophthalmic Epidemiol. 2016;23(sup1):39–45. doi: 10.1080/09286586.2016.1238945 . 27846369 10.1080/09286586.2016.1238945 PMC5706969

[39] Taleo F, Macleod CK, Marks M, Sokana O, Last A, Willis R, et al Integrated Mapping of Yaws and Trachoma in the Five Northern-Most Provinces of Vanuatu. PLoS Negl Trop Dis. 2017;11(1):e0005267 doi: 10.1371/journal.pntd.0005267 ; PubMed Central PMCID: PMCPMC5261559. 28118354 10.1371/journal.pntd.0005267 PMC5261559

[40] Bio AA, Boko PM, Dossou YA, Tougoue JJ, Kabore A, Sounouvou I, et al Prevalence of Trachoma in Northern Benin: Results from 11 Population-Based Prevalence Surveys Covering 26 Districts. Ophthalmic Epidemiol. 2017:1–9. doi: 10.1080/09286586.2017.1279337 . 28441120 10.1080/09286586.2017.1279337 PMC6837864

[41] Thylefors B, Dawson CR, Jones BR, West SK, Taylor HR. A simple system for the assessment of trachoma and its complications. Bull World Health Organ. 1987;65(4):477–83. ; PubMed Central PMCID: PMCPMC2491032. 3500800 PMC2491032

[42] WHO, UNICEF. Progress on sanitation and drinking-water—2013 update. Geneva, Switzerland: 2013.

[43] Cairncross S, Feachem RG. Environmental health engineering in the tropics: an introductory text. 2 ed: John Wiley & Sons Ltd; 1993.

[44] Blakely TA, Woodward AJ. Ecological effects in multi-level studies. Journal of epidemiology and community health. 2000;54(5):367–74. doi: 10.1136/jech.54.5.367 ; PubMed Central PMCID: PMC1731678. 10814658 10.1136/jech.54.5.367 PMC1731678

[45] Susser M. The logic in ecological: I. The logic of analysis. American journal of public health. 1994;84(5):825–9. ; PubMed Central PMCID: PMC1615050. 8179056 10.2105/ajph.84.5.825 PMC1615050

[46] Zou G. A modified poisson regression approach to prospective studies with binary data. Am J Epidemiol. 2004;159(7):702–6. . 15033648 10.1093/aje/kwh090

[47] Habtamu E, Wondie T, Aweke S, Tadesse Z, Zerihun M, Zewdie Z, et al Trachoma and Relative Poverty: A Case-Control Study. PLoS Negl Trop Dis. 2015;9(11):e0004228 doi: 10.1371/journal.pntd.0004228 ; PubMed Central PMCID: PMCPMC4657919. 26600211 10.1371/journal.pntd.0004228 PMC4657919

[48] Taylor HR, Burton MJ, Haddad D, West S, Wright H. Trachoma. Lancet. 2014;384(9960):2142–52. doi: 10.1016/S0140-6736(13)62182-0 . 25043452 10.1016/S0140-6736(13)62182-0

[49] Freeman MC, Garn JV, Sclar GD, Boisson S, Medlicott K, Alexander KT, et al The impact of sanitation on infectious disease and nutritional status: A systematic review and meta-analysis. Int J Hyg Environ Health. 2017;220(6):928–49. doi: 10.1016/j.ijheh.2017.05.007 . 28602619 10.1016/j.ijheh.2017.05.007

[50] Khandekar R, Thanah TTK, Thi PD. Impact of face washing and environmental improvement on reduction of active trachoma in Vietnam—a public health intervention study. Ophthalmic Epidemiology. 2006;13(1):43–52. doi: 10.1080/09286580500477507 16510346 10.1080/09286580500477507

[51] Emerson PM, Lindsay SW, Alexander N, Bah M, Dibba S-M, Faal HB, et al Role of flies and provision of latrines in trachoma control: cluster-randomised controlled trial. The Lancet. 2004;363(9415):1093–8. doi: 10.1016/s0140-6736(04)15891-1 10.1016/S0140-6736(04)15891-1 15064026

[52] Abdou A, Munoz BE, Nassirou B, Kadri B, Moussa F, Baarè I, et al How much is not enough? A community randomized trial of a Water and Health Education programme for Trachoma and Ocular C. trachomatis infection in Niger. Tropical Medicine & International Health. 2010;15(1):98–104. 10.1111/j.1365-3156.2009.02429.x PMC2867063 20409284

[53] Stoller NE, Gebre T, Ayele B, Zerihun M, Assefa Y, Habte D, et al Efficacy of latrine promotion on emergence of infection with ocular Chlamydia trachomatis after mass antibiotic treatment: a cluster-randomized trial. Int Health. 2011;3(2):75–84. doi: 10.1016/j.inhe.2011.03.004 ; PubMed Central PMCID: PMCPMC3139980. 21785663 10.1016/j.inhe.2011.03.004 PMC3139980

[54] Ejere HO, Alhassan MB, Rabiu M. Face washing promotion for preventing active trachoma. Cochrane Database Syst Rev. 2015;(2):CD003659 doi: 10.1002/14651858.CD003659.pub4 ; PubMed Central PMCID: PMCPMC4441394. 25697765 10.1002/14651858.CD003659.pub4 PMC4441394

[55] Rabiu M, Alhassan MB, Ejere HO, Evans JR. Environmental sanitary interventions for preventing active trachoma. Cochrane Database Syst Rev. 2012;(2):CD004003 doi: 10.1002/14651858.CD004003.pub4 ; PubMed Central PMCID: PMCPMC4422499. 22336798 10.1002/14651858.CD004003.pub4 PMC4422499

[56] Clasen T, Boisson S, Routray P, Torondel B, Bell M, Cumming O, et al Effectiveness of a rural sanitation programme on diarrhoea, soil-transmitted helminth infection, and child malnutrition in Odisha, India: a cluster-randomised trial. Lancet Glob Health. 2014;2(11):e645–53. doi: 10.1016/S2214-109X(14)70307-9 . 25442689 10.1016/S2214-109X(14)70307-9

[57] UN General Assembly, editor Transforming our world: the 2030 Agenda for Sustainable Development. United Nations: General Assembly; 2015.

[58] O'Loughlin R, Fentie G, Flannery B, Emerson PM. Follow-up of a low cost latrine promotion programme in one district of Amhara, Ethiopia: characteristics of early adopters and non-adopters. Trop Med Int Health. 2006;11(9):1406–15. doi: 10.1111/j.1365-3156.2006.01689.x . 16930263 10.1111/j.1365-3156.2006.01689.x

[59] WHO, UNICEF. Progress on drinking water and sanitation-2014 update. Geneva, Switzerland: World Health Organization and UNICEF, 2014.

[60] Garn JV, Sclar GD, Freeman MC, Penakalapati G, Alexander KT, Brooks P, et al The impact of sanitation interventions on latrine coverage and latrine use: A systematic review and meta-analysis. Int J Hyg Environ Health. 2016 doi: 10.1016/j.ijheh.2016.10.001 . 27825597 10.1016/j.ijheh.2016.10.001 PMC5414716

