# Supplementary material for: Sanitation and water supply coverage thresholds associated with active trachoma: Modeling cross-sectional data from 13 countries
Source: PLoS Negl Trop Dis. 2018 Jan 22;12(1):e0006110. doi: 10.1371/journal.pntd.0006110 (PMC5800679; doi:10.1371/journal.pntd.0006110)
Supplement: S1 Checklist — (DOC) [file pntd.0006110.s001.doc]

STROBE Statement—Checklist of items that should be included in reports of ***cross-sectional studies***

|  | Item No | Recommendation | Section |
| --- | --- | --- | --- |
| **Title and abstract** | 1 | (*a*) Indicate the study’s design with a commonly used term in the title or the abstract | Title: “modeling cross-sectional data from 13 countries” |
|  |  | (*b*) Provide in the abstract an informative and balanced summary of what was done and what was found | Abstract: “Methods and findings” section. |
| Introduction | | |  |
| Background/rationale | 2 | Explain the scientific background and rationale for the investigation being reported | Introduction: paragraphs 1 and 2 |
| Objectives | 3 | State specific objectives, including any prespecified hypotheses | Introduction: paragraph 3 |
| Methods | | |  |
| Study design | 4 | Present key elements of study design early in the paper | Methods: first three paragraphs |
| Setting | 5 | Describe the setting, locations, and relevant dates, including periods of recruitment, exposure, follow-up, and data collection | Methods: “Study context” section. “Study population” section. “Data collection and follow-up timeline” section. |
| Participants | 6 | (*a*) Give the eligibility criteria, and the sources and methods of selection of participants | Methods: “Study population” section.” “Households were sampled using…” “Fig 1. Study flow diagram.” |
| Variables | 7 | Clearly define all outcomes, exposures, predictors, potential confounders, and effect modifiers. Give diagnostic criteria, if applicable | Methods: “Outcomes” paragraph. “Predictors” paragraph. |
| Data sources/ measurement | 8* | For each variable of interest, give sources of data and details of methods of assessment (measurement). Describe comparability of assessment methods if there is more than one group | Methods: “Outcomes” paragraph. “Predictors” paragraph.  “Data analysis” section. |
| Bias | 9 | Describe any efforts to address potential sources of bias | Methods: second to last and third to last paragraphs are both on sensitivity analyses. |
| Study size | 10 | Explain how the study size was arrived at | Methods: “Fig 1. Study flow diagram” and “Study population” paragraph. |
| Quantitative variables | 11 | Explain how quantitative variables were handled in the analyses. If applicable, describe which groupings were chosen and why | Methods: “Predictors” paragraph. “Data analysis” section. |
| Statistical methods | 12 | (*a*) Describe all statistical methods, including those used to control for confounding | Methods: “Data analysis” section. Statistical models are shown in this section. |
|  |  | (*b*) Describe any methods used to examine subgroups and interactions |  |
|  |  | (*c*) Explain how missing data were addressed | Methods: “Data analysis” section. Statistical models explicitly show interactions of interest. |
|  |  | (*d*) If applicable, describe analytical methods taking account of sampling strategy | Methods: “*Data collection and follow-up timeline”* section describes sampling. |
|  |  | (*e*) Describe any sensitivity analyses | Methods: second to last and third to last paragraphs are both on sensitivity analyses. |
| Results | | |  |
| Participants | 13* | (a) Report numbers of individuals at each stage of study—eg numbers potentially eligible, examined for eligibility, confirmed eligible, included in the study, completing follow-up, and analysed | Methods: “Fig 1. Study flow diagram” and “Study population” paragraph. |
|  |  | (b) Give reasons for non-participation at each stage | Methods: “Fig 1. Study flow diagram” and “Study population” paragraph. |
|  |  | (c) Consider use of a flow diagram | Methods: “Fig 1. Study flow diagram” |
| Descriptive data | 14* | (a) Give characteristics of study participants (eg demographic, clinical, social) and information on exposures and potential confounders | Results: First paragraph. Table 1. |
|  |  | (b) Indicate number of participants with missing data for each variable of interest | Methods: “Fig 1. Study flow diagram” |
| Outcome data | 15* | Report numbers of outcome events or summary measures | Results: First paragraph. Table 1.*“*% with TF” |
| Main results | 16 | (*a*) Give unadjusted estimates and, if applicable, confounder-adjusted estimates and their precision (eg, 95% confidence interval). Make clear which confounders were adjusted for and why they were included | Results: Figs 2-5. Table 2, surrounding text, and supplementary tables. |
|  |  | (*b*) Report category boundaries when continuous variables were categorized | Results: Table 2. Figs 2-5. These are shown in left-most columns of tables and in x-axes of figures. |
|  |  | (*c*) If relevant, consider translating estimates of relative risk into absolute risk for a meaningful time period | n/a |
| Other analyses | 17 | Report other analyses done—eg analyses of subgroups and interactions, and sensitivity analyses | Supplementary Figs S1-S8. Tables S1-S6. |
| Discussion | | |  |
| Key results | 18 | Summarise key results with reference to study objectives | Discussion: first paragraph |
| Limitations | 19 | Discuss limitations of the study, taking into account sources of potential bias or imprecision. Discuss both direction and magnitude of any potential bias | Discussion: second paragraph |
| Interpretation | 20 | Give a cautious overall interpretation of results considering objectives, limitations, multiplicity of analyses, results from similar studies, and other relevant evidence | Discussion: Third and fourth paragraph |
| Generalisability | 21 | Discuss the generalisability (external validity) of the study results | Methods: Second to last paragraph. “Our main analyses used data from all 13 countries with the goal to improve generalizability.” |
| Other information | | |  |
| Funding | 22 | Give the source of funding and the role of the funders for the present study and, if applicable, for the original study on which the present article is based | Included separately in submission system. |

*Give information separately for exposed and unexposed groups.

**Note:** An Explanation and Elaboration article discusses each checklist item and gives methodological background and published examples of transparent reporting. The STROBE checklist is best used in conjunction with this article (freely available on the Web sites of PLoS Medicine at http://www.plosmedicine.org/, Annals of Internal Medicine at http://www.annals.org/, and Epidemiology at http://www.epidem.com/). Information on the STROBE Initiative is available at www.strobe-statement.org.
