# Supplementary figures and images for: Sanitation and water supply coverage thresholds associated with active trachoma: Modeling cross-sectional data from 13 countries
Source: PLoS Negl Trop Dis. 2018 Jan 22;12(1):e0006110. doi: 10.1371/journal.pntd.0006110 (PMC5800679; doi:10.1371/journal.pntd.0006110)

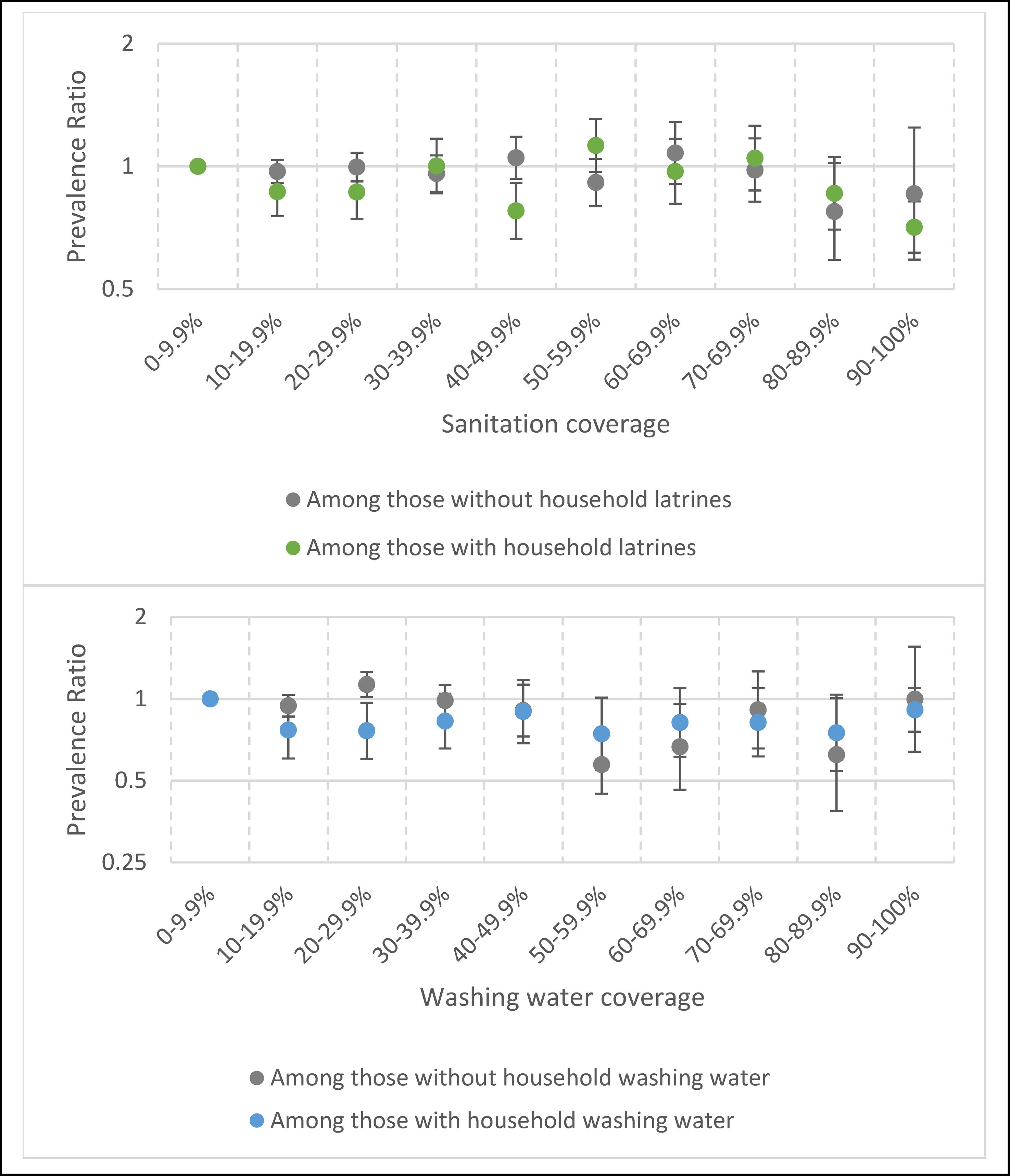

Supplement: S1 Fig — (TIF) [file pntd.0006110.s002.tif]

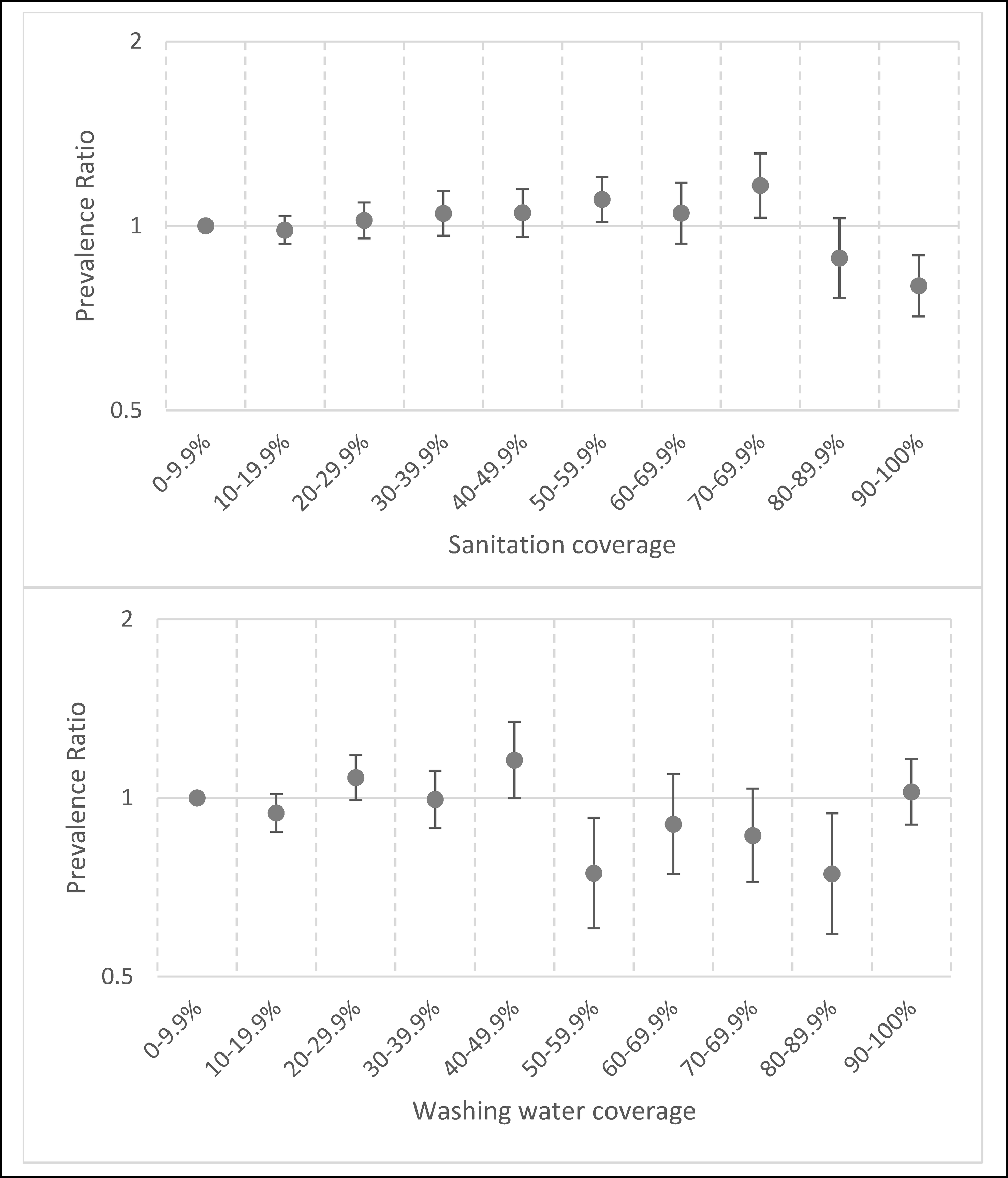

Supplement: S2 Fig — (TIF) [file pntd.0006110.s003.tif]

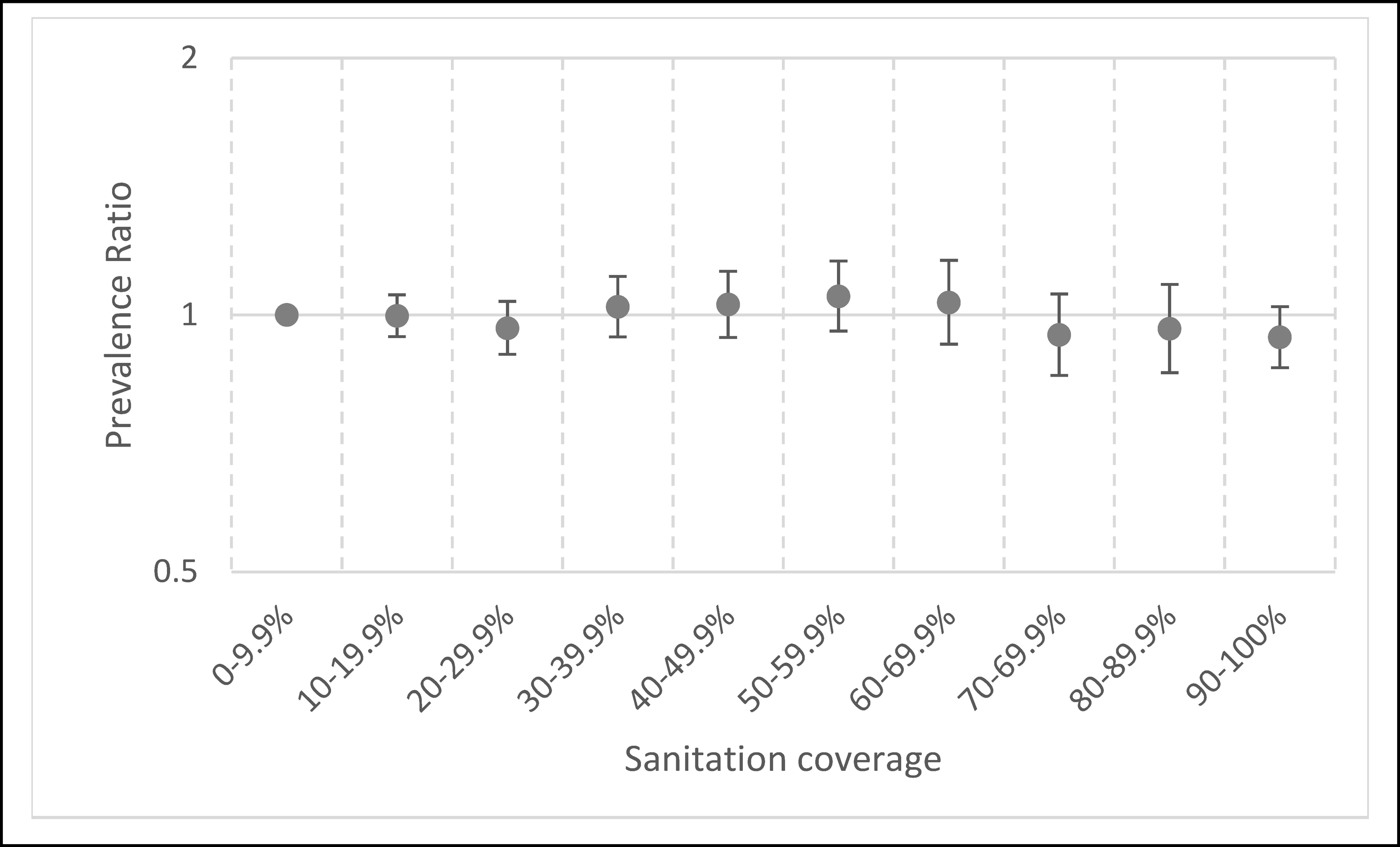

Supplement: S3 Fig — (TIF) [file pntd.0006110.s004.tif]

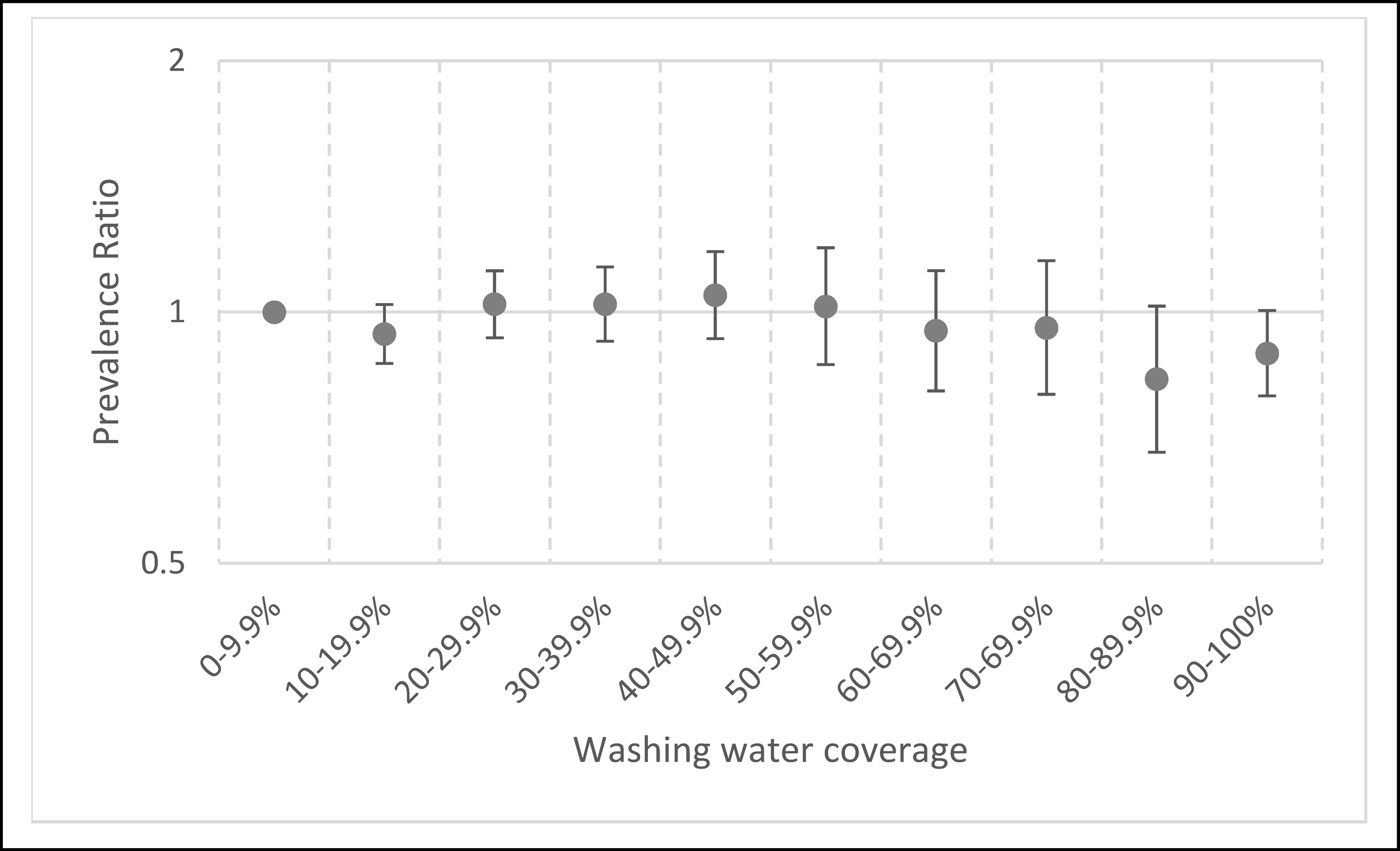

Supplement: S4 Fig — (TIF) [file pntd.0006110.s005.tif]

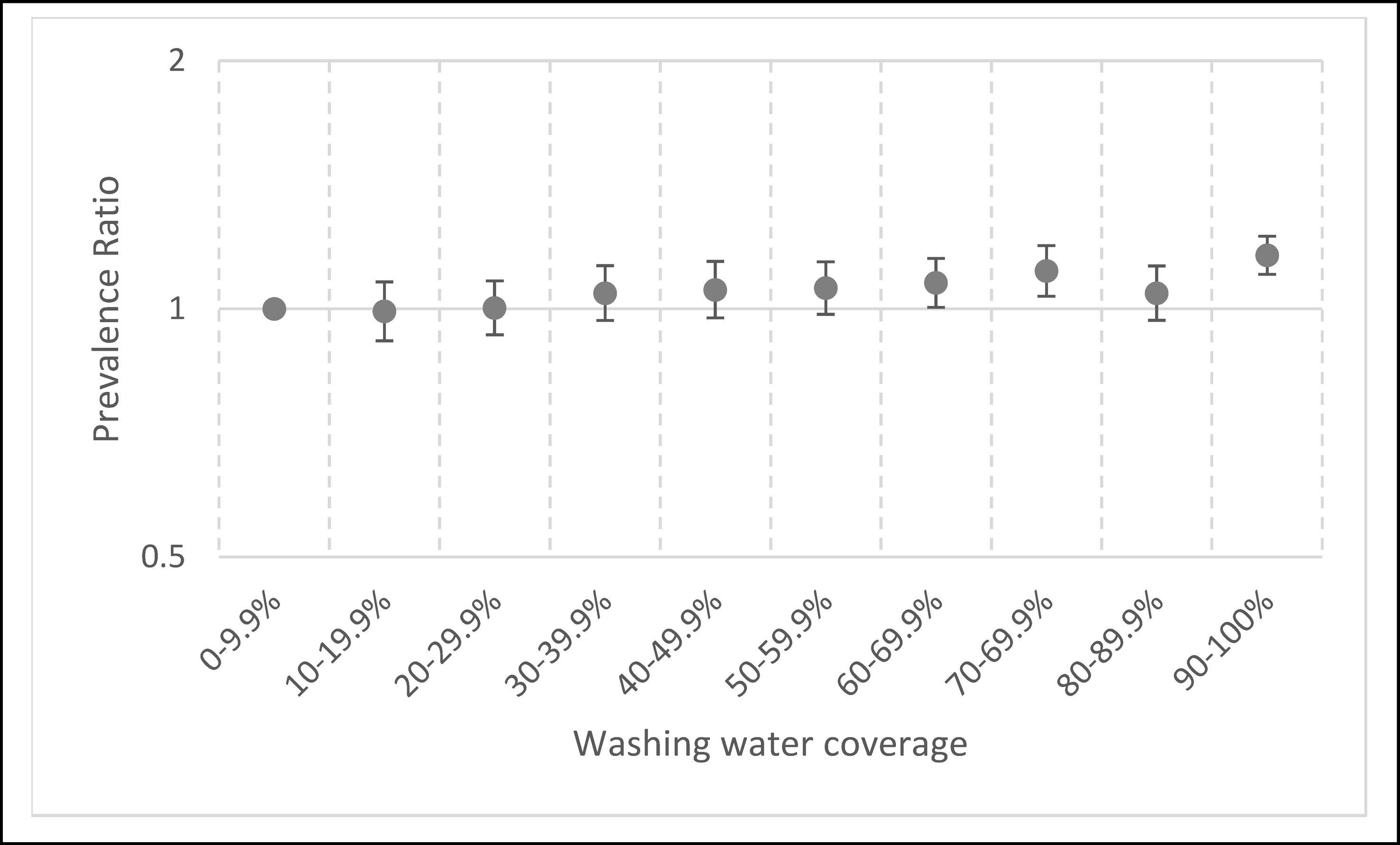

Supplement: S5 Fig — (TIF) [file pntd.0006110.s006.tif]

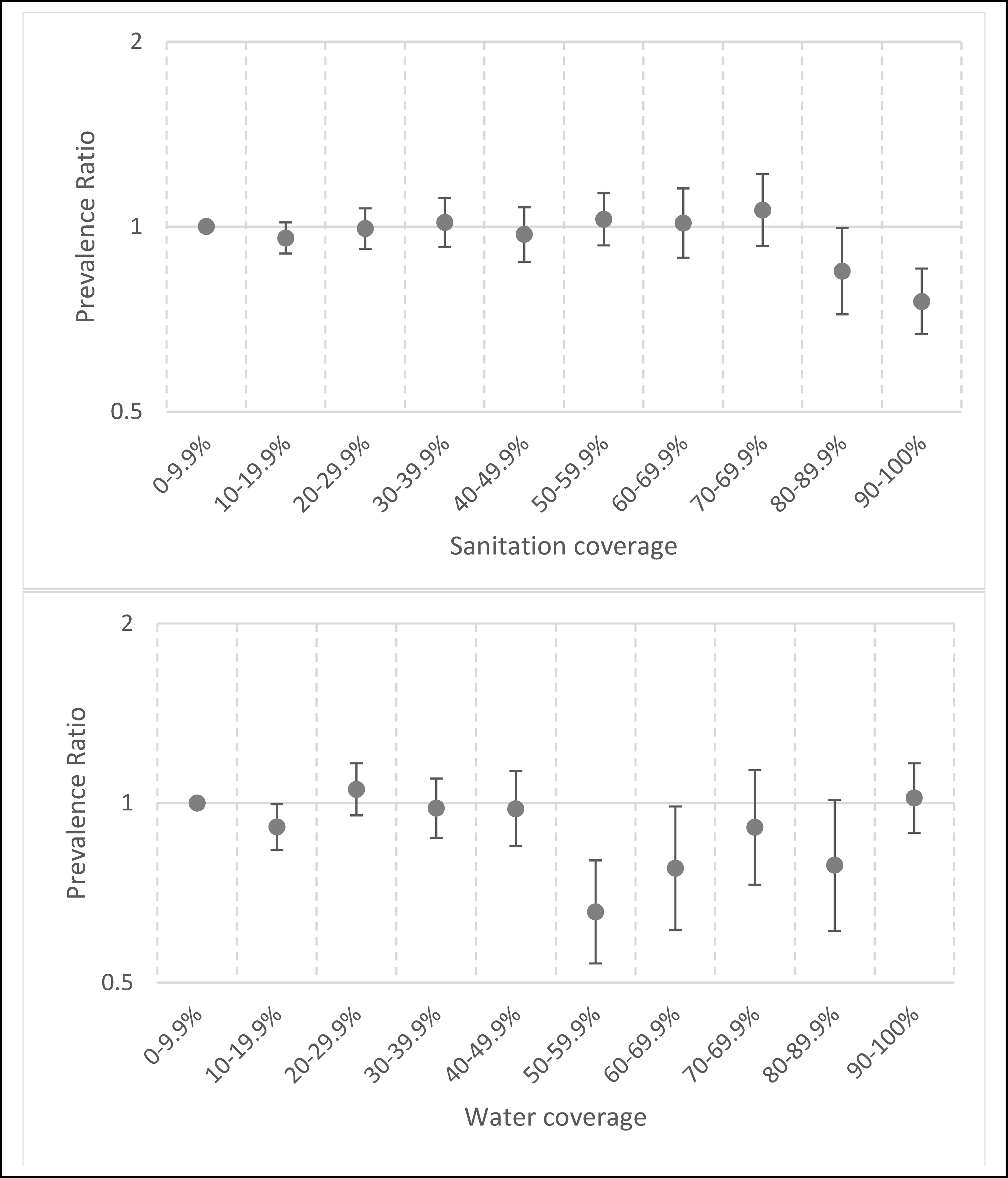

Supplement: S6 Fig — (TIF) [file pntd.0006110.s007.tif]

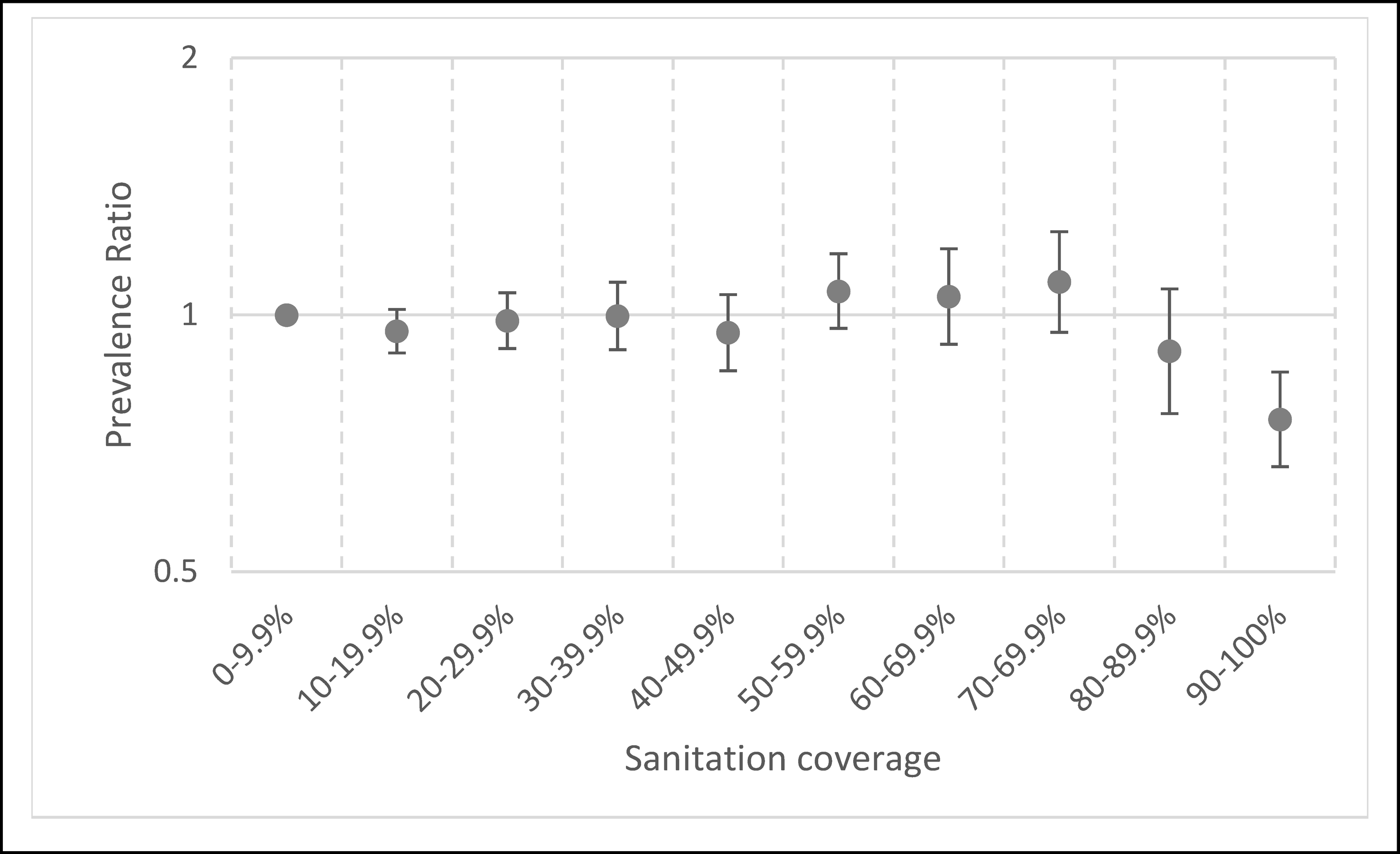

Supplement: S7 Fig — (TIF) [file pntd.0006110.s008.tif]

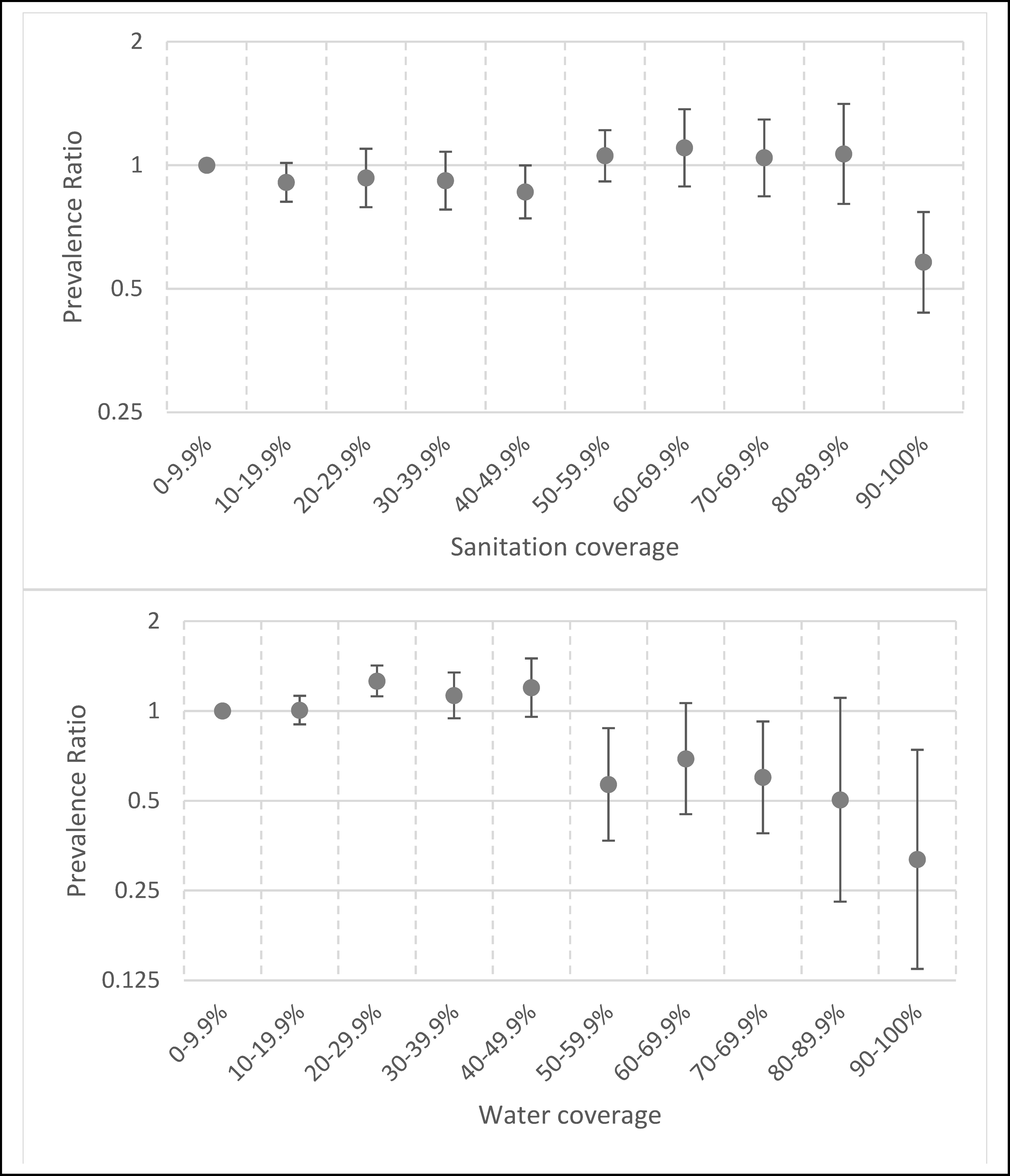

Supplement: S8 Fig — (TIF) [file pntd.0006110.s009.tif]
