# Supplementary material for: Sanitation and water supply coverage thresholds associated with active trachoma: Modeling cross-sectional data from 13 countries
Source: PLoS Negl Trop Dis. 2018 Jan 22;12(1):e0006110. doi: 10.1371/journal.pntd.0006110 (PMC5800679; doi:10.1371/journal.pntd.0006110)
Supplement: S1 Table — (DOCX) [file pntd.0006110.s010.docx]

Table S1. Descriptive results for trachomatous inflammation—follicular for all ages.

|  |  | N participants | % with TF^a^ (%SE)^b^ |
| --- | --- | --- | --- |
| Total |  | 2,019,085 | 4.4 (0.1) |
| By country |  |  |  |
| Côte d'Ivoire |  | 38,706 | 5.8 (0.3) |
| Egypt |  | 10,266 | 7.2 (0.7) |
| Guinea |  | 45,324 | 2.6 (0.2) |
| Malawi |  | 74,826 | 3 (0.2) |
| Yemen |  | 123,040 | 1.8 (0.1) |
| Nigeria |  | 775,286 | 2.1 (0.1) |
| Vanuatu |  | 3,428 | 5.4 (1.1) |
| Ethiopia |  | 478,410 | 9.3 (0.1) |
| Lao People's Democratic Republic |  | 21,572 | 1 (0.1) |
| Solomon Islands |  | 9,213 | 9.6 (0.8) |
| Democratic Republic of the Congo |  | 142,366 | 6 (0.2) |
| Mozambique |  | 206,626 | 1.9 (0.1) |
| Benin |  | 90,022 | 6.5 (0.7) |

^a^ Trachomatous inflammation—follicular in either or both eyes. ^b^ We accounted for clustering in the standard error estimates.
