# Supplementary material for: Sanitation and water supply coverage thresholds associated with active trachoma: Modeling cross-sectional data from 13 countries
Source: PLoS Negl Trop Dis. 2018 Jan 22;12(1):e0006110. doi: 10.1371/journal.pntd.0006110 (PMC5800679; doi:10.1371/journal.pntd.0006110)
Supplement: S2 Table — (DOCX) [file pntd.0006110.s011.docx]

Table S2. Multivariable model showing the household-level and community-level associations between improved sanitation, water and trachomatous inflammation—follicular (TF) among all ages.

|  | Prevalence ratio (95% CI)^a^ |
| --- | --- |
| *Sanitation variables* |  |
| Community sanitation coverage (%)^b, c^ |  |
| 0-9.9% | ref |
| 10-19.9% | 0.98 (0.93, 1.04) |
| 20-29.9% | 1.02 (0.95, 1.09) |
| 30-39.9% | 1.05 (0.96, 1.14) |
| 40-49.9% | 1.05 (0.96, 1.15) |
| 50-59.9% | 1.10 (1.01, 1.20)** |
| 60-69.9% | 1.05 (0.94, 1.18) |
| 70-69.9% | 1.16 (1.03, 1.31)** |
| 80-89.9% | 0.89 (0.76, 1.03) |
| 90-100% | 0.80 (0.71, 0.90)** |
| Household-level sanitation (yes vs. no)^b^ | 0.87 (0.83, 0.91)** |
| *Water variables* |  |
| Community washing water coverage (%)^c, d^ |  |
| 0-9.9% | ref |
| 10-19.9% | 0.94 (0.88, 1.02) |
| 20-29.9% | 1.08 (0.99, 1.18)* |
| 30-39.9% | 0.99 (0.89, 1.11) |
| 40-49.9% | 1.16 (1.00, 1.34) |
| 50-59.9% | 0.75 (0.60, 0.93)** |
| 60-69.9% | 0.90 (0.74, 1.10) |
| 70-69.9% | 0.86 (0.72, 1.04) |
| 80-89.9% | 0.75 (0.59, 0.94)** |
| 90-100% | 1.02 (0.90, 1.16) |
| Household-level washing water (yes vs. no)^d^ | 0.86 (0.79, 0.93)** |
| Other included confounders not shown^a^ | .^a^ |

***** = significant at 0.1 level; ****** = significant at 0.05 level. ^a^ The model controlled for all variables shown in the table and additionally controlled for country, prevalence of TF in the cluster, participant’s age and participant’s sex; the model included a random effect to account for clustering. ^b^Improved sanitation, as defined by the JMP (WHO and UNICEF 2013). ^c^ Community-level results are also shown graphically in Fig. S1. ^d^ Improved water, as defined by the JMP (WHO and UNICEF 2013), but with an additional constraint that the water source had to be located in the residence/yard.
