# Supplementary material for: Sanitation and water supply coverage thresholds associated with active trachoma: Modeling cross-sectional data from 13 countries
Source: PLoS Negl Trop Dis. 2018 Jan 22;12(1):e0006110. doi: 10.1371/journal.pntd.0006110 (PMC5800679; doi:10.1371/journal.pntd.0006110)
Supplement: S3 Table — (DOCX) [file pntd.0006110.s012.docx]

Table S3. Multivariable model showing the household-level and community-level associations between *any type of latrine (vs. no latrine/open defecation),* washing water and trachomatous inflammation—follicular (TF) among ages 1-9.

|  | Prevalence ratio (95% CI)^a^ |
| --- | --- |
| *Sanitation variables* |  |
| Community sanitation coverage (%)^b, c^ |  |
| 0-9.9% | ref |
| 10-19.9% | 1.00 (0.94, 1.06) |
| 20-29.9% | 0.97 (0.90, 1.04) |
| 30-39.9% | 1.02 (0.94, 1.11) |
| 40-49.9% | 1.03 (0.94, 1.13) |
| 50-59.9% | 1.05 (0.96, 1.16) |
| 60-69.9% | 1.03 (0.92, 1.16) |
| 70-69.9% | 0.95 (0.85, 1.06) |
| 80-89.9% | 0.96 (0.86, 1.09) |
| 90-100% | 0.94 (0.87, 1.02) |
| Household-level sanitation (yes vs. no)^b^ | 0.86 (0.83, 0.91)** |
| *Water variables* |  |
| Community washing water coverage (%)^c, d^ |  |
| 0-9.9% | ref |
| 10-19.9% | 0.92 (0.85, 1.01)* |
| 20-29.9% | 1.07 (0.97, 1.18) |
| 30-39.9% | 1.00 (0.89, 1.12) |
| 40-49.9% | 0.97 (0.84, 1.12) |
| 50-59.9% | 0.65 (0.53, 0.80)** |
| 60-69.9% | 0.80 (0.62, 1.01)* |
| 70-69.9% | 0.91 (0.73, 1.13) |
| 80-89.9% | 0.78 (0.61, 1.01)* |
| 90-100% | 0.96 (0.83, 1.09) |
| Household-level washing water (yes vs. no)^d^ | 0.82 (0.75, 0.89)** |
| Other included confounders not shown^a^ | .^a^ |

***** = significant at 0.1 level; ****** = significant at 0.05 level. ^a^ The model controlled for all variables shown in the table and additionally controlled for country, prevalence of TF in the cluster, participant’s age and participant’s sex; the model included a random effect to account for clustering. ^b^Any sanitation *vs*. open defecation/no sanitation. ^c^ Community-level results are also shown graphically in Fig. S2. ^d^ Improved water, as defined by the JMP (WHO and UNICEF 2013), but with an additional constraint that the water source had to be located in the residence/yard.
