# Supplementary material for: Sanitation and water supply coverage thresholds associated with active trachoma: Modeling cross-sectional data from 13 countries
Source: PLoS Negl Trop Dis. 2018 Jan 22;12(1):e0006110. doi: 10.1371/journal.pntd.0006110 (PMC5800679; doi:10.1371/journal.pntd.0006110)
Supplement: S6 Table — (DOCX) [file pntd.0006110.s015.docx]

Table S6. Multivariable model showing the household-level and community-level associations between improved sanitation*,* washing water and trachomatous inflammation—follicular (TF) *and/or trachomatous inflammation—intense (TI)* among ages 1-9.

|  | Prevalence ratio (95% CI)^a^ |
| --- | --- |
| *Sanitation variables* |  |
| Community sanitation coverage (%)^b, c^ |  |
| 0-9.9% | ref |
| 10-19.9% | 0.96 (0.90, 1.02) |
| 20-29.9% | 0.99 (0.92, 1.07) |
| 30-39.9% | 1.02 (0.93, 1.11) |
| 40-49.9% | 0.97 (0.88, 1.08) |
| 50-59.9% | 1.03 (0.93, 1.13) |
| 60-69.9% | 1.01 (0.89, 1.15) |
| 70-69.9% | 1.06 (0.93, 1.22) |
| 80-89.9% | 0.85 (0.72, 1.00)** |
| 90-100% | 0.76 (0.67, 0.85)** |
| Household-level sanitation (yes vs. no)^b^ | 0.89 (0.85, 0.93)** |
| *Water variables* |  |
| Community washing water coverage (%)^c, d^ |  |
| 0-9.9% |  |
| 10-19.9% | 0.91 (0.83, 1.00)** |
| 20-29.9% | 1.05 (0.95, 1.17) |
| 30-39.9% | 0.98 (0.87, 1.10) |
| 40-49.9% | 0.98 (0.85, 1.13) |
| 50-59.9% | 0.66 (0.54, 0.80)** |
| 60-69.9% | 0.78 (0.61, 0.99)** |
| 70-69.9% | 0.91 (0.73, 1.14) |
| 80-89.9% | 0.79 (0.61, 1.01)* |
| 90-100% | 1.02 (0.89, 1.17) |
| Household-level washing water (yes vs. no)^d^ | 0.80 (0.74, 0.87)** |
| Other included confounders not shown^a^ | .^a^ |

***** = significant at 0.1 level; ****** = significant at 0.05 level. ^a^ The model controlled for all variables shown in the table and additionally controlled for country, prevalence of TF and/or TI in the cluster, participant’s age and participant’s sex; the model included a random effect to account for clustering. ^b^Improved sanitation, as defined by the JMP (WHO and UNICEF 2013). ^c^ These community-level results are also shown graphically in Fig. S5. ^d^ Improved water, as defined by the JMP (WHO and UNICEF 2013), but with an additional constraint that the water source had to be located in the residence/yard.
