# Supplementary material for: Sanitation and water supply coverage thresholds associated with active trachoma: Modeling cross-sectional data from 13 countries
Source: PLoS Negl Trop Dis. 2018 Jan 22;12(1):e0006110. doi: 10.1371/journal.pntd.0006110 (PMC5800679; doi:10.1371/journal.pntd.0006110)
Supplement: S1 Text — (PDF) [file pntd.0006110.s016.pdf]

Demo_3_RESIDENT_v4 (eng)

Variable Name	Question Text	Saved Value
startTime	Hidden from user	Timestamp of form open
endTime	Hidden from user	Timestamp of form save
ResEU	EU	User entered text
ResCluster	Cluster	User entered text
ResHouseholdID	Enter head of household or household ID code	User entered text
Name	Name	User entered text
Sex	Sex	1 Male<br>2 Female
Age	Age (years)	User entered integer
Examined	Examined?	1 Yes (with consent)<br>2 Absent<br>3 Refused<br>4 Other
RightEyeTT	TT	0 Sign absent<br>1 Sign present<br>2 Not able to grade
OfferedSurgeryRightEye	Have you ever been offered surgery by a health worker to correct the trichiasis (in-turned eyelashes) in this eye?	1 Yes, a health worker informed me and offered me surgery, and I had surgery.<br>2 Yes, a health worker informed me and offered me surgery and I accepted the offer, but I have not yet had surgery.<br>3 Yes, a health worker informed me and offered me surgery, but I declined it.<br>0 No health worker informed me and offered me surgery.<br>8 Don't know
OfferedEpiRightEye	Have you ever been offered epilation by a health workers to correct the trichiasis (in-turned eyelashes) in	0 No

	this eye?	1 Yes<br>8 Don't know
ScarringRightEye	Does this eye show TS?	0 Sign absent<br>1 Sign present<br>2 Unable to evert the eyelid
RightEyeTF	TF	0 Sign absent<br>1 Sign present<br>2 Not able to grade
RightEyeTI	TI	0 Sign absent<br>1 Sign present<br>2 Not able to grade
LeftEyeTT	TT	0 Sign absent<br>1 Sign present<br>2 Not able to grade
OfferedSurgeryLeftEye	Have you ever been offered surgery by a health worker to correct the trichiasis (in-turned eyelashes) in this eye?	1 Yes, a health worker informed me and offered me surgery, and I had surgery.<br>2 Yes, a health worker informed me and offered me surgery and I accepted the offer, but I have not yet had surgery.<br>3 Yes, a health worker informed me and offered me surgery, but I declined it.<br>0 No health worker informed me and offered me surgery.<br>8 Don't know
OfferedEpiLeftEye	Have you ever been offered epilation by a health workers to correct the trichiasis (in-turned eyelashes) in this eye?	0 No<br>1 Yes<br>8 Don't know

ScarringLeftEye	Does this eye show TS?	0 Sign absent<br>1 Sign present<br>2 Unable to evert the eyelid
LeftEyeTF	TF	0 Sign absent<br>1 Sign present<br>2 Not able to grade
LeftEyeTI	TI	0 Sign absent<br>1 Sign present<br>2 Not able to grade
ResNotes	Additional notes?	User entered text
meta	Hidden from user	
instanceID	Hidden from user	
