# Supplementary material for: Sanitation and water supply coverage thresholds associated with active trachoma: Modeling cross-sectional data from 13 countries
Source: PLoS Negl Trop Dis. 2018 Jan 22;12(1):e0006110. doi: 10.1371/journal.pntd.0006110 (PMC5800679; doi:10.1371/journal.pntd.0006110)
Supplement: S2 Text — (PDF) [file pntd.0006110.s017.pdf]

Demo_2_HOUSEHOLD_v3 (eng)

Variable Name	Question Text	Saved Value
startTime	Hidden from user	Timestamp of form open
endTime	Hidden from user	Timestamp of form save
GPS	Capture GPS data	User captured location coordinates
HouseholdInterviewer	Recorder ID (4 numbers)	User entered text
HouseholdEU	EU	User entered text
HouseholdCluster	Cluster	User entered text
HouseholdID	Enter head of household or household ID code	User entered text
W1DrinkSource	In the dry season, what is the main source of drinking-water for members of your household?	1 Piped water into dwelling<br>2 Piped water into yard/plot<br>3 Public tap/standpipe<br>4 Tubewell/borehole<br>5 Protected dug well<br>6 Unprotected dug well<br>7 Protected spring<br>8 Unprotected spring<br>9 Rainwater collection<br>10 Water vendor<br>11 Surface water (e.g. river, dam, lake, canal)<br>99 Other (specify next)
W1DrinkSourceSpecify	Specify	User entered text
W2GetDrinkWater	How long does it take to go there, get water, and come back?	1 Water source in the yard<br>2 Less than 30 minutes<br>3 Between 30 minutes and 1 hour<br>4 More than 1 hour
W3WashWater	In the dry season, what is the main source of water used by your household for washing faces?	1 Piped water into dwelling<br>2 Piped water into yard/pot<br>3 Public tap/standpipe

		4 Tubewell/borehole<br>5 Protected dug well<br>6 Unprotected dug well<br>7 Protected spring<br>8 Unprotected spring<br>9 Rainwater collection<br>10 Water vendor<br>11 Surface water (e.g. river, dam, lake, canal)<br>99 Other (specify next)
W3WashSourceSpecify	Specify	User entered text
W4GetWashWater	If you collected water there to bring back to the house, how long does it take to go there, get water, and come back?	0 All face washing done at the water source<br>1 Water source in the yard<br>2 Less than 30 minutes<br>3 Between 30 minutes and 1 hour<br>4 More than 1 hour
S1Defecate	Where do you and other adults in the household usually defecate?	1 Shared latrine<br>2 Private latrine<br>3 No structure, outside near the house<br>4 No structure, in the bush or field<br>9 Other
S2SeeLatrine	Observation: Ask to see the latine/toilet	1 Flush/pour flush to piped sewer system<br>2 Flush/pour flush to septic tank<br>3 Flush/pour flush to pit latrine<br>4 Flush/pour flush to open

		drains<br>5 Flush/pour flush to unknown place<br>6 Ventilated improved pit latrine (VIP)<br>7 Pit latrine with slab<br>8 Pit latrine without slab/open pit<br>9 Composting toilet<br>10 Bucket<br>11 Hanging toilet/hanging latrine<br>12 No facilities or bush or field<br>99 Other (specify next)
S2SeeLatrineSpecify	Specify	User entered text
H1Distance	Observation	0 No<br>1 Yes<br>5 Not applicable (no latrine/toilet)
H2Water	Observation	0 No<br>1 Yes<br>5 Not applicable (no latrine/toilet)
H3Soap	Observation	0 No<br>1 Yes<br>5 Not applicable (no latrine/toilet)
meta	Hidden from user	
instanceID	Hidden from user	
