# Supplementary material for: Sanitation and water supply coverage thresholds associated with active trachoma: Modeling cross-sectional data from 13 countries
Source: PLoS Negl Trop Dis. 2018 Jan 22;12(1):e0006110. doi: 10.1371/journal.pntd.0006110 (PMC5800679; doi:10.1371/journal.pntd.0006110)
Supplement: S3 Text — (PDF) [file pntd.0006110.s018.pdf]

Supplemental text S3.

Researchers are welcome to request access to the de-identified datasets, through the contacts below:

Côte d'Ivoire	Kouakou IIunga Marie Madeleine magdy_koua@yahoo.fr
Egypt	Khaled Amer amerk888@gmail.com
Guinea	André Goepogui agoep@yahoo.fr
Malawi	Khumbo Kalua khumbokalua@yahoo.com
Yemen	Tawfik Al-Khatib tawfik234@yahoo.com
Nigeria	Nicholas Olobio olobio@yahoo.com
Ethiopia	Biruck Kebede biruck1@gmail.com
Lao PDR	Khamphoua Southisombath southi1961@gmail.com
Solomon Islands	Oliver Sokana osokana@moh.gov.sb
DRC	Jean Ndjemba drndjemba@yahoo.fr
Mozambique	Mariamo Saide Abdala Mbofana mariamoabdala@yahoo.com.br
Benin	Wilfrid Batcho wbatcho@yahoo.fr
Vanuatu	Fasihah Taleo taleof@who.int
